# GLM-based optimization of NGS data analysis: A case study of Roche 454, Ion Torrent PGM and Illumina NextSeq sequencing data

**DOI:** 10.1371/journal.pone.0171983

**Published:** 2017-02-21

**Authors:** Sarah Sandmann, Aniek O. de Graaf, Bert A. van der Reijden, Joop H. Jansen, Martin Dugas

**Affiliations:** 1 Institute of Medical Informatics, University of Münster, Münster, Germany; 2 Laboratory Hematology, RadboudUMC, Nijmegen, Netherlands; Mayo Clinic Arizona, UNITED STATES

## Abstract

**Background:**

There are various next-generation sequencing techniques, all of them striving to replace Sanger sequencing as the gold standard. However, false positive calls of single nucleotide variants and especially indels are a widely known problem of basically all sequencing platforms.

**Methods:**

We considered three common next-generation sequencers—Roche 454, Ion Torrent PGM and Illumina NextSeq—and applied standard as well as optimized variant calling pipelines. Optimization was achieved by combining information of 23 diverse parameters characterizing the reported variants and generating individually calibrated generalized linear models. Models were calibrated using amplicon-based targeted sequencing data (19 genes, 28,775 bp) from seven to 12 myelodysplastic syndrome patients. Evaluation of the optimized pipelines and platforms was performed using sequencing data from three additional myelodysplastic syndrome patients.

**Results:**

Using standard analysis methods, true mutations were missed and the obtained results contained many artifacts—no matter which platform was considered. Analysis of the parameters characterizing the true and false positive calls revealed significant platform- and variant specific differences. Application of optimized variant calling pipelines considerably improved results. 76% of all false positive single nucleotide variants and 97% of all false positive indels could be filtered out. Positive predictive values could be increased by factors of 1.07 to 1.27 in case of single nucleotide variant calling and by factors of 3.33 to 53.87 in case of indel calling. Application of the optimized variant calling pipelines leads to comparable results for all next-generation sequencing platforms analyzed. However, regarding clinical diagnostics it needs to be considered that even the optimized results still contained false positive as well as false negative calls.

## Introduction

Personalized medicine is a concept that exhibits great potential for many diseases. An important aspect of this concept is the identification of genomic variants like single nucleotide variants (SNVs) and indels in patient samples that enable optimization of diagnosis, prognosis and eventually therapy.

A few years ago when Sanger sequencing [1] was still the gold standard for genome sequencing, turnaround time as well as cost of sequencing was high [2]. However, things have changed since various next-generation sequencing (NGS) techniques have been launched in recent years. Sequencing, especially targeted sequencing, can now be performed consuming a fraction of time and costs [2–4].

However, NGS is not free from flaws. Sequencing errors, leading to false positive calls of SNVs and particularly indels, are a widespread problem for basically all NGS platforms [5–9]. For establishing the use of NGS data in clinical routine, though, high sensitivity as well as a low false positive rate are equally required.

It would therefore be useful to investigate every platforms’ possibilities on the basis of standard as well as individually optimized analysis approaches. Until now, many different filters optimizing the output of a single sequencing platform have been reported [8–13]. These filters usually include one or two parameters characterizing the called variants and a set of unchangeable thresholds. However, these approaches do not account for differences in data generated by the same sequencing platform due to specific sequencing set up (e.g. target enrichment, library preparation or laboratory conditions). Furthermore, it often remains unclear why the presented parameters and thresholds were chosen in a particular way. Although they fit the analyzed training data set best, it remains to be seen whether this is also true for other data.

In the case study we present, we (1) compare the performance with respect to sensitivity and positive predictive value (PPV) of three common NGS platforms—Roche 454 Genome Sequencer FLX [2], Ion Torrent PGM [14] and Illumina NextSeq [15]. We consider both SNVs and indels on the basis of a standard analysis pipeline using GATK [16] for variant calling. Diagnostic material from 10 to 15 myelodysplastic syndrome (MDS) patients forms the basis of the analysis. To investigate inter-platform variation, the same nine patients are sequenced on every platform. To investigate intra-platform variation, five patients are sequenced a second time on the same platform.

Subsequently, we (2) analyze a set of 23 diverse parameters characterizing the reported variants with respect to their importance on separating true from false positive calls. An individual analysis is performed for the three platforms as well as for SNVs and indels. This approach enables a detailed investigation of the different platforms’ characteristics.

Finally, we (3) develop and apply optimized variant calling approaches. Based on a training data set, consisting of 12 to 17 datasets per platform, individually calibrated generalized linear models (GLMs) are estimated. In general, GLMs are a frequently used tool in relation to medical as well as biological trials (e.g. [17] or [18]). In the context of our case study, the models provide an advanced filtration strategy for the output generated by GATK, aiming at improving PPV and retaining sensitivity. A test dataset consisting of three additional, independent datasets per platform is analyzed for model validation.

## Materials and methods

### Standard analysis pipeline without GLM

**Fig 1:**
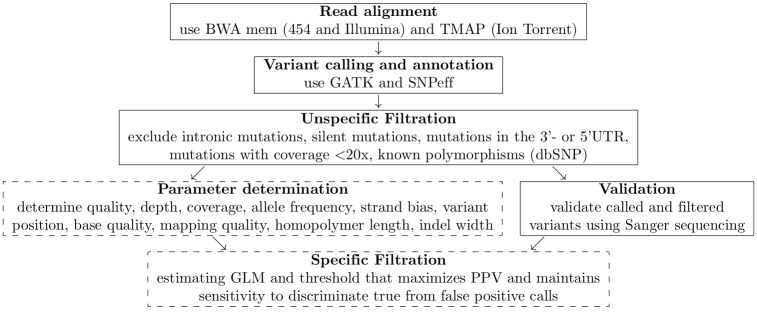
Overview of the variant calling pipeline (steps marked by dashed frames are only performed in case of the variant calling pipeline with GLM).

The analysis pipeline was basically the same for all platforms that were evaluated as well as for the different variants that were called. Fig 1 (steps in continuous frames only) gives an overview of the standard analysis pipeline (training- and test data set).

Read alignment was performed using BWA [19]. In case of 454 and Illumina NextSeq sequencing data the alignment algorithm was invoked using BWA mem. In case of Ion Torrent sequencing data, TMAP [20] (http://github.com/iontorrent/tmap) was used. Variant calling was performed using GATK [16]. SNPeff [21] was used for annotation. Unspecific filtration of the called variants was performed. Intronic variants, silent variants, variants in the 3’-or 5’-UTR, variants with less than 20x coverage and known polymorphisms according to dbSNP [22] were excluded.

The remaining calls were categorized as either true or false by two independent experts. A selection of calls—assumed true and false, as well as SNVs and indels—was confirmed by Sanger sequencing of the original patient material.

### Parameters and their importance

For all variants passing the *Unspecific Filtration*, a set of parameters (see Table 1, see section S5 Appendix for details) was determined using R [23] (http://www.R-project.org).

**Table 1:** Overview of the parameters investigated for the variant calling pipeline with GLM.

Category	Parameter	Origin	SNVs	Indels
Quality and depth	*Q*	vcf file (QUAL)	x	x
	*DP*	vcf file (DP)	x	x
	*QD*	vcf file (QD)	x	x
Coverage	*Cov*_*total*	calculated	x	x
	*Cov*_*ref*	calculated	x	x
	*Cov*_*vcf*	vcf file (AD: #ref+#alt)	x	x
Allele frequency	*AF*_*total*	calculated	x	x
	*AF*_*ref*	calculated	x	x
	*AF*_*vcf*	vcf file (AD: #alt/(#ref+#alt))	x	x
Strand bias	*SB*	calculated	x	x
	*SB*_*vcf*	vcf file (FS)	x	x
	*SOR*	vcf file (SOR)	x	x
Variant position	*VP*	calculated	x	x
	*VP*_*vcf*	vcf file (ReadPosRankSum)	x	x
Base quality	*BQ*	calculated	x	
	*BQ*_*vcf*	vcf file (BaseQRankSum)	x	x
Mapping quality	*MQ*	vcf file (MQ)	x	x
	*MQRank*	vcf file (MQRankSum)	x	x
Homopolymer length	*HP*	calculated		x
	*HP*_*AT*	calculated		x
	*HP*_*CG*	calculated		x
Indel width	*VARW*	calculated ([8])		x
	*DevGT*	calculated		x

Against the background of separating true positive calls from artifacts, the relative variable importance (RVI) [24] of every parameter was calculated (see script S2 Script). GLMs containing correlated parameters (e.g. *Q* and *QD*) were not considered as this would bias Akaike’s Information Criterion (AIC) and thus also the RVI. Furthermore, not converging models were not considered either. However, excluding models from consideration also results in an imbalance of the number of models that contain each variable. This imbalance directly influences the RVI as well. Therefore, we perform a normalization of the results. The normalized RVI of a parameter *p* gets calculated by $RVI\left(p\right)=RVI_{raw}\left(p\right)\cdot \#models\left(p\right)/\sum_{i=1}^{n}\#models\left(i\right)$ with *RVI*_*raw*_(*p*) being the raw RVI of *p*, #*models*(*p*) being the number of considered models containing *p* and $\sum_{i=1}^{n}\#models\left(i\right)$ being the total number of considered models.

### Optimized analysis pipeline with GLM

In addition to the standard analysis pipeline, an optimized pipeline was applied (see Fig 1, steps in continuous- and dashed frames). This pipeline combines standard variant calling with the previously determined parameters.

The actual optimization of the variant calling pipeline was performed in the last step. The determined parameters as well as the information from the validation were used to estimate GLMs. We selected the model that was best in separating true variants from the false positives that were called in the datasets. Parameter selection was performed using forward selection and AIC (see section S7 Appendix and scripts S3 and S4 Scripts). Subsequently, a threshold was estimated taking into account three conditions: (1) No true positive call is mistaken as false positive, i.e. sensitivity is maintained. (2) As many false positive calls as possible are identified as such. (3) A threshold as low as possible, fulfilling conditions (1) and (2), is chosen.

The above described pipeline was used in case of the training dataset. A similar pipeline was used for the analysis of the test dataset. The only difference was in the *Specific Filtration*. As the GLMs and the thresholds had already been estimated on the basis of the training datasets, these steps were not repeated. Instead, the estimated models and thresholds from the training dataset were applied.

### Analyzed data

To evaluate the three NGS platforms, sequencing data from diagnostic material of patients with MDS were investigated. The study was approved by MEC: Medisch Ethische Toetsingscommissie (METc; Medical Ethical Committee) of the Vrije Universiteit Medisch Centrum (VUmc; VU University Medical Center Amsterdam), contact person: C.M.M. Licht, PhD, address: BS7, kamer H-565, PO Box 7057, 1007 MB Amsterdam, The Netherlands (EudraCT nr.: 2008-002195-10). All participants provided written consent to participate in this study. The ethics committee approved this consent procedure.

Bone marrow aspirates were taken from patients at diagnosis. The samples were taken by research nurses. From one aliquot of each bone marrow sample, the mononuclear cells were selected and DNA was extracted using NucleoSpin DNA isolation kits (Macherey-Nagel, Germany) according to the instructions of the manufacturer. Details on how DNA was prepared is described in section S1 Appendix.

An overview of the sequenced samples is given in Table 2. Amplicon-based targeted sequencing of 19 genes (28,775 bp) known to be recurrently mutated in MDS was performed on each subject and platform (see S1 Table). Sequencing was performed as described in section S1 Appendix. Sequencing data is available at https://uni-muenster.sciebo.de/index.php/s/GlTYWTt0Bcyqa8f.

**Table 2:** Overview of the subjects sequenced on 454, Ion Torrent and Illumina NextSeq (comparison set marked with a *c*, re-sequencing set marked with an *r*).

Sample	454		Ion Torrent		Illumina	
	1st set	2nd set	1st set	2nd set	1st set	2nd set
UPN001	x^*c*^		x^*c*,*r*^	x^*r*^	x^*c*^	
UPN002	x^*c*^		x^*c*,*r*^	x^*r*^	x^*c*^	
UPN003	x^*c*^		x^*c*,*r*^	x^*r*^	x^*c*^	
UPN004	x^*c*^		x^*c*,*r*^	x^*r*^	x^*c*^	
UPN005	x^*c*^		x^*c*,*r*^	x^*r*^	x^*c*^	
UPN006	x^*c*^		x^*c*^		x^*c*^	
UPN007	x^*c*^		x^*c*^		x^*c*^	
UPN008	x^*c*^		x^*c*^		x^*c*^	
UPN009	x^*c*,*r*^	x^*r*^	x^*c*^		x^*c*^	
UPN010	x^*r*^	x^*r*^				
UPN011	x^*r*^	x^*r*^				
UPN012	x^*r*^	x^*r*^				
UPN013	x^*r*^	x^*r*^				
UPN014					x^*r*^	x^*r*^
UPN015					x^*r*^	x^*r*^
UPN016					x^*r*^	x^*r*^
UPN017					x^*r*^	x^*r*^
UPN018					x^*r*^	x^*r*^
UPN019	x				x	
UPN020	x		x			

To investigate inter-platform variation, a subset of nine samples (UPN001-UPN009) was sequenced on every platform (= *comparison subset*; samples marked with a *c* in Table 2).

To investigate intra-platform variation, for each platform DNA from five subjects was amplified and sequenced a second time, using exactly the same primers and conditions (= *re-sequencing subset*; samples marked with an *r* in Table 2).

### Training- and test subset

To derive an optimized analysis pipeline, data are randomly divided into two subsets: a training subset and a test subset. For 454, the training subset consists of samples UPN001-UPN007, UPN009-UPN013 (1st set) and UPN009-UPN013 (2nd set). The test subset consists of samples UPN008, UPN019 and UPN020. For Ion Torrent, the training subset consists of samples UPN001-UPN007 (1st set) and UPN001-UPN005 (2nd set). The test subset consists of samples UPN008, UPN009 and UPN20. Regarding Illumina the training subset consists of samples UPN001-UPN007, UPN014-UPN018 (1st set) and UPN014-UPN018 (2nd set) The test subset consists of samples UPN008, UPN009 and UPN019.

## Results

### Data quality

Data derived from NGS techniques can show a lot of variation. Differences can be due to the principle sequencing chemistry and methods of different platforms, each with its distinct error profile. The expected read length depends on the type of platform and the sequencing mode or kit that is chosen. Different sets of primers were designed for each sequencing technology to meet these requirements. There were slight differences in target regions.

An intersecting target region covering 28,775 bp could be defined that was common to the datasets generated by the three sequencing platforms (see S2 Table). A fair comparison of the different sequencing platforms is only possible if the intersecting target region is sufficiently covered by all samples.

Regarding the comparison subset, in all but one case (Ion Torrent, UPN002) the rate of target bases covered at least once was higher than 95%. 23 out of 27 samples even feature 50x coverage of more than 90% of the targeted bases (see S3–S5 Tables).

**Fig 2:**
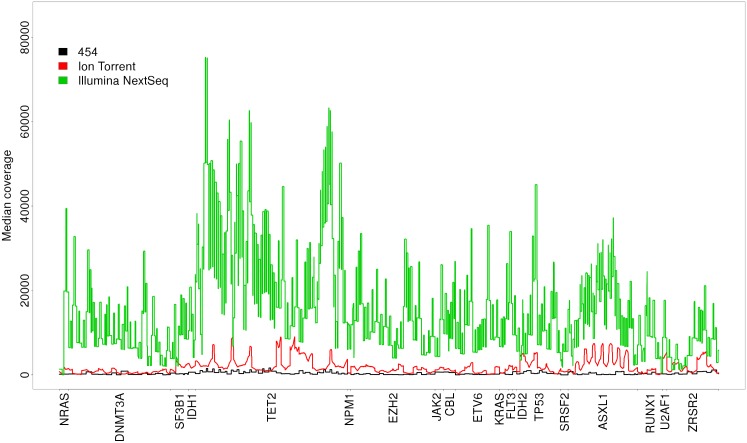
Median coverage of the genes in the intersecting target region in the case of 454 (black), Ion Torrent (red) and Illumina (green) considering the comparison data set.

The coverage plot in Fig 2 shows the median coverage across the intersecting target region for all sequencing platforms. The coverage of the targeted bases was very uneven—not only when comparing different platforms, but also for different genes in the target region. For example for TET2 a highly uneven coverage profile is observed. 454 data generally featured the lowest coverage (median reads on target ${\tilde{x}}_{454}=45,335$), while Illumina NextSeq data featured the highest coverage (median reads on target ${\tilde{x}}_{Illumina}=3,879,811$; ${\tilde{x}}_{IonT}=553,816$). However, the sequencing set up that was chosen and the number of samples analyzed in parallel favours high coverage for Illumina samples.

S1 Fig shows that there are no bases completely uncovered in all samples, which is why a general coverage problem in e.g. GC rich regions [25] was not expected to bias the variant calling process. In case of Illumina NextSeq data, all bases in the intersecting target region are covered by all samples analyzed.

Analysis of the other samples’ coverages showed comparable results (see S3–S5 Tables).

### Calling SNVs

#### Standard analysis pipeline without GLM

For the single nucleotide variants (SNVs), it is essential to distinguish between true and false positive calls. Categorization, which includes manual inspection of all 54 calls, was performed by two independent biological and bioinformatical experts. Exemplary true as well as false positive calls were validated using Sanger sequencing (45% of the true positives, 43% of the false positives; see S1 File for details). Table 3 sums up information on sensitivity and the PPV with respect to the comparison subset, the re-sequencing subset and all data. Sensitivity should be regarded with restriction as data may contain unknown false negative variants. However, as all samples were sequenced two to six times and analyzed twice using different approaches (see section S8 Appendix), it seems apt to assume that the vast majority of present mutations was indeed detected.

**Table 3:** True- and false positive SNV calls, sensitivity and PPV considering the comparison subset (*n* = 9), the re-sequencing subset (*n* = 5) and all data (454 and Illumina: *n* = 15, Ion Torrent: *n* = 10), using the standard analysis pipleine (without GLM). Only those variants are considered that are covered by at least 20 reads.

Dataset	Platform		SNVs	False Positives	Sensitivity	PPV
Comparison	454		11	3	0.84	0.79
	Ion Torrent		12	6	0.92	0.67
	Illumina NextSeq		11	1	0.84	0.92
Re-sequencing	454	Set1	14	1	1.00	0.93
		Set2	12	0	0.86	1.00
		Overlap	12	0	0.86	1.00
	Ion Torrent	Set1	4	6	0.80	0.40
		Set2	4	1	0.80	0.80
		Overlap	4	0	0.80	0.80
	Illumina NextSeq	Set1	8	2	0.89	0.80
		Set2	9	0	1.00	1.00
		Overlap	8	0	0.89	1.00
Altogether	454		40	4	0.91	0.91
	Ion Torrent		21	7	0.91	0.75
	Illumina NextSeq		29	3	0.88	0.91

The SNV calling results—according to the standard analysis pipeline without GLM—indicate that the three sequencing platforms perform almost equally well. The majority of variants was detected. However, no platform succeeded in calling all variants (*sens*_454_ = 0.91, *sens*_*IonT*_ = 0.91, *sens*_*Illumina*_ = 0.88). A diverse set of variants was missing in case of each platform. Three out of four mutations missed in the 454 sequencing data are present in the raw variant calling results, but they are filtered out by GATK due to bad quality and low coverage. One out of four mutations missed in the Illumina NextSeq is automatically filtered out by GATK. None of the mutations missed in the Ion Torrent data are present in the raw variant calling results.

In addition to the false negative calls, all platforms report false positive calls. In case of both 454- and Illumina NextSeq sequencing data, 9% of the calls were false positives. 25% of all calls reported for the Ion Torrent data were false positives, which suggested a slightly better performance of 454 and Illumina NextSeq when using a standard analysis pipeline.

Samples UPN001 to UPN009 were sequenced on every sequencing platform (comparison subset) to allow for the investigation of inter-platform variation. Data indicates a slightly better performance of Ion Torrent when considering sensitivity (*sens*_454_ = 0.84, *sens*_*IonT*_ = 0.92, *sens*_*Illumina*_ = 0.84). However, with respect to PPV, Ion Torrent performs worst, while Illumina NextSeq has the best results (*PPV*_454_ = 0.79, *PPV*_*IonT*_ = 0.67 and *PPV*_*Illumina*_ = 0.92).

A detailed analysis of the missed calls points out that the platforms usually differ in the calls they miss (see S1 File). One SNV is missed by Ion Torrent and Illumina (UPN003: chr21:36,206,893 G>A), while another SNV is missed by 454 and Illumina (UPN008: chr4:106,197,302 G>A).

Altogether, analysis of the comparison subset indicates a slight advantage in favor of Illumina NextSeq, when calling SNVs with a standard analysis pipeline.

Five samples were sequenced twice on 454, Ion Torrent and Illumina NextSeq (re-sequencing subset). Re-sequencing the same samples on the same platform points out the general variation in sensitivity and PPV. While Ion Torrent data shows the smallest variation with respect to sensitivity (*sens*_*IonT*_ = 0.80 for set1 and set2), it shows the greatest variation with respect to PPV (set1: *PPV*_*IonT*_ = 0.40; set2: *PPV*_*IonT*_ = 0.80).

If the variant calling results of both sequencing sets are combined by looking at the overlapping calls, the number of false positive calls is zero for all sequencing platforms. This observation indicates that the false positive calls resulted from random- and not systematic errors.

#### Parameters and their importance

For all parameters characterizing SNVs, their relative variable importance with respect to separating true from false positive calls is determined. The results are summed up in Table 4.

**Table 4:** Normalized relative variable importance for all parameters characterizing SNVs, considering 454, Ion Torrent and Illumina NextSeq sequencing data.

Parameter	454	Ion Torrent	Illumina
*Q*	**2.04**	0.60	**4.12**
*DP*	0.58	0.61	0.29
*QD*	1.73	0.81	0
*Cov*_*total*	1.01	1.00	**1.15**
*Cov*_*ref*	1.01	1.00	1.14
*Cov*_*vcf*	0.81	0.55	0.32
*AF*_*total*	0.71	0.78	0
*AF*_*ref*	0.74	0.78	0
*AF*_*vcf*	1.00	0.69	0
*SB*	0.38	0.17	0.44
*SB*_*vcf*	0.85	0.70	2.12
*SOR*	0.80	1.26	0.56
*VP*	0.67	0.63	0.41
*VP*_*vcf*	**0.93**	**0.83**	0.73
*BQ*	0.84	0.73	0.66
*BQ*_*vcf*	2.91	0.80	1.08
*MQ*	1.07	0.76	1.20
*MQRank*	0.45	0.84	0.30

Differences in the RVI can be observed when comparing parameters as well as platforms. Regarding 454 data, the base quality parameter reported in the vcf files, *BQ*_*vcf*, features the greatest importance when separating true from false positive calls. It is interesting to observe that *BQ*, which is a parameter characterizing base quality calculated by us, has a considerably lower relative variable importance (0.84). Further parameters of great importance are *Q*, *QD* and *MQ*.

Thoroughly different results are obtained when analyzing Ion Torrent data. Although *BQ*_*vcf* still features a high RVI (0.80), *SOR*, a parameter characterizing the strand bias, has the greatest RVI (1.26). However, it seems unlikely that strand bias in general features a great importance when separating true from false positive SNVs as both, *SB* and *SB*_*vcf*, show low values of RVI (0.17 and 0.70). Apart from *SOR*, the parameters *Cov*_*total* and *Cov*_*ref* are of great importance according to RVI.

Due to its low number of false positive calls, a majority of models estimated on the basis of Illumina data, do not converge (for details see S6 Table). Any model containing one of the parameters *QD*, *AF*_*total*, *AF*_*ref* or *AF*_*vcf* never converges. Therefore, the relative variable importance of these parameters is zero. *Q*, the quality value determined by GATK, features the greatest relative variable importance by far (4.12). Further parameters of great importance are *SB*_*vcf*, *MQ*, *Cov*_*total* and *Cov*_*ref*.

#### Optimized analysis pipeline with GLM

In addition to the standard analysis pipeline, an individual, optimized pipeline is investigated for every sequencing platform. The optimized pipeline, including filtration of the results by the help of a GLM, is derived from the training subset and validated using the test subset. The results for both subsets, comparing the standard approach (without GLM) with the optimized approach (with GLM), are summed up in Table 5.

**Table 5:** True- and false positive SNV calls, sensitivity (sens) and PPV considering the training subset (454 and Illumina: *n* = 12, Ion Torrent: *n* = 7) and the test subset (*n* = 3), comparing the standard analysis pipleine (without GLM) and the optimized analysis pipleine (with GLM). Only those variants are considered that are covered by at least 20 reads.

Dataset	Platform	SNVs without GLM				SNVs with GLM			
		SNVs	False Positives	Sens	PPV	SNVs	False Positives	Sens	PPV
Training	454	36	3	0.92	0.92	36	1	0.92	0.97
	Ion Torrent	15	7	0.88	0.68	15	1	0.88	0.94
	Illumina NextSeq	27	3	0.90	0.90	27	1	0.90	0.96
Test	454	4	1	0.80	0.80	4	0	0.80	1.00
	Ion Torrent	6	0	1.00	1.00	6	0	1.00	1.00
	Illumina NextSeq	2	0	0.67	1.00	2	0	0.67	1.00

Combining the information on the SNVs that were called in the training datasets, three GLMs—one for each platform—were estimated returning a probability ${\hat{p}}_{i}$ for an SNV being a true positive. The linear predictors ${\hat{\eta }}_{i\_SNV\_454}$, ${\hat{\eta }}_{i\_SNV\_IonT}$ and ${\hat{\eta }}_{i\_SNV\_Illumina}$ leading to the best results were defined as follows:

$$\begin{array}{c} {\hat{\eta }}_{i\_SNV\_454}=-7.08+0.02\cdot x_{i\_Q}+6.70\cdot x_{i\_VP\_vcf} \end{array}$$ (1)

$$\begin{array}{c} {\hat{\eta }}_{i\_SNV\_IonT}=-4.58+20.62\cdot x_{i\_VP\_vcf} \end{array}$$ (2)

$$\begin{array}{c} {\hat{\eta }}_{i\_SNV\_Illumina}=9.09+0.01\cdot x_{i\_Q}-0.02\cdot x_{i\_Cov\_total} \end{array}$$ (3)

The previously performed RVI analysis already indicated a different error profile of the three sequencing platforms compared. This thesis is further supported by the observation that all models feature a different set of covariates (for detailed information on the covariates see S7 Table). The value of the AIC was comparable in case of all models (454: 10.75; Ion Torrent: 9.09; Illumina NextSeq: 12.01), indicating a similar performance.

Regarding the RVI, it can be observed that the applied forward selection led to the inclusion of parameters with a high relative variable importance (see bold entries in Table 4). Comparing the RVIs of the selected parameters, *Q* (quality) features a considerably greater relative variable importance compared to *VP*_*vcf* (variant position) in case of ${\hat{\eta }}_{i\_SNV\_454}$ (2.04 compared to 0.93). *Q* also features a greater relative variant importance compared to *Cov*_*total* (coverage) in case of ${\hat{\eta }}_{i\_SNV\_Illumina}$ (4.12 compared to 1.15).

S6 Fig shows that the GLMs successfully assigned a high probability to most of the true positive SNVs, while they assigned a low probability to the majority of the false positive SNVs. Thresholds (*p*_*SNV*_454_ = 0.28, *p*_*SNV*_*IonT*_ = 0.04 and *p*_*SNV*_*Illumina*_ = 0.15) were chosen to retain the original sensitivity and to reduce the number of false positive calls to a maximum extent. The last column in Table 5 shows the change in PPV if filtration by the estimated GLMs is performed. An improvement could be observed for all sequencing platforms. The improvement was most considerable in case of the Ion Torrent sequencing data. Regarding the calling of SNVs in the training dataset, the different sequencing platforms now performed equally well considering sensitivity as well as the number of false positive calls.

To test the performance of the model approach on an independent dataset, the test subset was analyzed. In case of the 454 sequencing data the application of the optimized analysis pipeline improved the results. Sensitivity was not harmed, while the false positive call was successfully filtered out. No change could be observed in case of Ion Torrent and Illumina NextSeq. Data did not contain any false positive calls. The individually calibrated GLMs correctly identified all true positive SNVs.

On the basis of the test subset, Ion Torrent showed the highest sensitivity, both using a standard and an optimized analysis pipeline.

### Calling indels

#### Standard analysis pipeline without GLM

Analogous to the analysis of the SNVs, an analysis of the called indels was performed. All 890 reported indels were categorized and manually inspected by two independent biological and bioinformatical experts as either true or false positive. Exemplary true as well as false positive calls were validated using Sanger sequencing (8% of the true positives, 0.005% of the false positives; see S1 File for details). Table 6 sums up information on sensitivity and the PPV with respect to the comparison subset, the re-sequencing subset and all data (see supplement for details).

**Table 6:** True- and false positive indel calls, sensitivity and PPV considering the comparison subset (*n* = 9), the re-sequencing subset (*n* = 5) and all data (454 and Illumina: *n* = 15, Ion Torrent: *n* = 10), using the standard analysis pipleine (without GLM). Only those variants are considered that are covered by at least 20 reads.

Dataset	Platform		Indels	False Positives	Sensitivity	PPV
Comparison	454		3	77	0.60	0.04
	Ion Torrent		5	422	1.00	0.01
	Illumina NextSeq		3	17	0.60	0.15
Re-sequencing	454	Set1	0	26	/	/
		Set2	0	75	/	/
		Overlap	0	17	/	/
	Ion Torrent	Set1	2	235	1.00	0.01
		Set2	2	297	1.00	0.01
		Overlap	2	123	1.00	0.02
	Illumina NextSeq	Set1	4	6	0.67	0.40
		Set2	5	11	1.00	0.31
		Overlap	4	4	0.67	0.5
Altogether	454		6	186	0.75	0.03
	Ion Torrent		8	800	1.00	0.01
	Illumina NextSeq		13	37	0.76	0.26

Considering all available data, it became obvious that all platforms again detected the majority of true positive indels. Ion Torrent even succeeded in detecting all true positives. However, the higher sensitivity of Ion Torrent was accompanied by an increased number of false positive calls and thus the lowest PPV (*PPV*_*IonT*_ = 0.01). The highest PPV could be observed in case of Illumina NextSeq (*PPV*_*Illumina*_ = 0.26).

An evaluation of inter-platform variation with respect to the platforms’ indel calling performance was done using nine samples (UPN001 to UPN009) that were sequenced on every sequencing platform. In the standard analysis pipeline Ion Torrent data featured greater sensitivity compared to the 454- and Illumina NextSeq data, detecting five out of five indels, instead of three. One indel is not called in both the 454- and Illumina NextSeq data (UPN007; chr2:25,463,545 delG). Regarding the number of false positive calls, Illumina NextSeq showed the best results by far (*PPV*_454_ = 0.04, *PPV*_*IonT*_ = 0.01 and *PPV*_*Illumina*_ = 0.15).

The fact that the comparison subset only contained five true positive indels makes it difficult to compare the performance. While Ion Torrent performed best with respect to sensitivity, Illumina NextSeq performed best with respect to PPV. However, the observed low sensitivity in case of Illumina (*PPV*_*Illumina*_ = 0.60) could be a random observation. The 422 false positive calls that were reported in case of Ion Torrent are on the contrary unlikely being a random observation. Thus, data does once again indicate a slight advantage in favor of Illumina NextSeq, when calling indels with a standard analysis pipeline.

The 454 sequencing data of the re-sequencing subset did not contain any true indels. Thus, neither sensitivity nor the PPV could be calculated.

Significant intra-platform specific variation was detected when comparing the number of false positive calls detected in the first and second set (see S1 File for details). In the second 454 set, 2.88 times as many false positive indels were called compared to the first set—although the same samples were analyzed. In the second Illumina NextSeq set, 1.83 times as many false positive indels were called compared to the first set. Regarding Ion Torrent data, an increase in the number of false positive calls of 26% could be observed comparing the first and second set.

Considering only those variants that were called in both re-sequencing sets led to a reduced number of false positive calls and a slightly decline in the number of true positives in case of Illumina NextSeq data. However, the number of remaining false positive calls was still relatively high for all sequencing platforms, indicating that a high percentage of sequencing errors was not due to random-, but to systematic errors of the sequencing technique itself, e.g. artifacts in homopolymeric regions.

#### Parameters and their importance

For all 22 parameters characterizing indels, their relative variable importance with respect to separating true from false positive calls is determined. The results are summed up in Table 7.

**Table 7:** Normalized relative variable importance for all parameters characterizing indels, considering 454, Ion Torrent and Illumina NextSeq sequencing data.

Parameter	454	Ion Torrent	Illumina
*Q*	0.54	0.74	**1.02**
*DP*	0.28	0.46	2.00
*QD*	**5.68**	2.06	0.42
*Cov*_*total*	0.32	**1.02**	0.63
*Cov*_*ref*	0.35	0.99	0.49
*Cov*_*vcf*	0.44	0.61	**1.39**
*AF*_*total*	2.90	0.73	0.95
*AF*_*ref*	1.42	0.61	0.58
*AF*_*vcf*	0.22	1.70	0.24
*SB*	0.27	0.00	1.02
*SB*_*vcf*	0.12	0.02	0.79
*SOR*	0.63	**4.42**	0.58
*VP*	0.19	1.30	1.74
*VP*_*vcf*	0.36	0.49	0.34
*BQ*_*vcf*	**0.26**	0.41	0.44
*MQ*	0.35	**1.65**	0.61
*MQRank*	0.39	0.70	0.76
*HP*	**3.16**	**2.59**	1.24
*HP*_*AT*	0.10	0.24	**0.76**
*HP*_*CG*	0.69	0.90	0.74
*VARW*	0.35	0.81	0.60
*DevGT*	0.74	0.56	1.87

Similar to the RVI analysis in case of SNVs, differences in the RVI can be observed when comparing parameters as well as platforms. *QD*, quality by depth, has the greatest relative variable importance (5.68) considering 454 data. Furthermore, *HP* and *AF*_*total* feature high RVI values, which indicates a great importance of these variables when separating true from false positive indels.

Regarding Ion Torrent data, *SOR* features the greatest RVI (4.42). This observation is remarkable as this parameter characterizing strand bias already featured the greatest importance when analyzing SNV calls. However, just like in case of the SNVs, *SB* and *SB*_*vcf*—both characterizing strand bias as well –, feature no or hardly any variable importance (0 and 0.02). Apart from *SOR*, *HP*, *QD*, *AF*_*vcf* and *MQ* feature great importance according to RVI.

Analysis of Illumina NextSeq data leads to considerably different results. Different from 454 or Ion Torrent data, the homopolymer length *HP* features a relatively low RVI (1.24). The parameter with the greatest importance in separating true from false positive indels is *DP*—the depth reported in the vcf file (2.00). Further important parameters are *DevGT*, *VP* and *Cov*_*vcf*.

#### Optimized analysis pipeline with GLM

In addition to the standard analysis pipeline, an individual, optimized pipeline is investigated for every NGS platform. Analogous to the SNVs, the optimized pipeline is derived from the training subset and validated using the test subset. Table 8 sums up the results for both subsets, comparing the standard analysis pipeline (without GLM) with the optimized analysis pipeline (with GLM).

**Table 8:** True- and false positive indel calls, sensitivity (sens) and PPV considering the training subset (454 and Illumina: *n* = 12, Ion Torrent: *n* = 7) and the test subset (*n* = 3), comparing the standard analysis pipleine (without GLM) and the optimized analysis pipleine (with GLM). Only those variants are considered that are covered by at least 20 reads.

Dataset	Platform	Indels without GLM				Indels with GLM			
		Indels	False Positives	Sens	PPV	Indels	False Positives	Sens	PPV
Training	454	3	158	0.60	0.02	3	4	0.60	0.43
	Ion Torrent	7	644	1.00	0.01	7	4	1.00	0.64
	Illumina NextSeq	12	31	0.80	0.28	12	2	0.80	0.86
Test	454	3	28	1.00	0.10	1	1	0.33	0.50
	Ion Torrent	1	156	1.00	0.01	1	3	1.00	0.25
	Illumina NextSeq	1	6	0.50	0.14	1	0	0.50	1.00

Using the information on the indels that were called in the training subsets in case of the three different sequencing platforms, three GLMs could be estimated returning a probability for an indel being a true positive. The linear predictors ${\hat{\eta }}_{i\_Indel\_454}$, ${\hat{\eta }}_{i\_Indel\_IonT}$ and ${\hat{\eta }}_{i\_Indel\_Illumina}$ leading to the best results were defined as follows:

$$\begin{array}{c} {\hat{\eta }}_{i\_Indel\_454}=27.06-31.02\cdot x_{i\_HP}+0.58\cdot x_{i\_QD}+16.69\cdot x_{i\_BQ\_vcf} \end{array}$$ (4)

$$\begin{array}{ccc} {\hat{\eta }}_{i\_Indel\_IonT}=-64.76-4.02\cdot x_{i\_SOR}-2.93\cdot x_{i\_HP}+0.81\cdot x_{i\_MQ}+ & & \\ 0.01\cdot x_{i\_Cov\_total} & \end{array}$$ (5)

$$\begin{array}{c} {\hat{\eta }}_{i\_Indel\_Illumina}=-12.09+0.11\cdot x_{i\_Cov\_vcf}-0.01\cdot x_{i\_Q}+2.51\cdot x_{i\_HP\_AT} \end{array}$$ (6)

Similar to the GLMs in the case of the SNVs, all models feature a different set of covariates (for detailed information on the covariates see S9 Table). However, it has to be noticed that the length of a homopolymer (HP) in which an indel is detected, is part of ${\hat{\eta }}_{i\_Indel\_454}$ as well as of ${\hat{\eta }}_{i\_Indel\_IonT}$, and features a great relative variable importance (3.16 and 2.59). This observation is in line with the knowledge that false positive indel calls are often located in homopolymeric regions as for 454- and Ion Torrent data and to a far lesser extent to Illumina data. *HP*_*AT*, which is part of the Illumina model, features a considerably smaller RVI (0.76). Furthermore, it has to be noted that the regression parameter is positive in this case. The presence of an indel call within a homopolymer of A’s or T’s thus increases the probability of being a true positive.

Comparing the parameters with the highest RVIs to those included in our models, shows, that a majority of covariates indeed feature a high or even the highest RVI values (see bold entries in Table 7). The only exception from this observation is *BQ*_*vcf* in case of ${\hat{\eta }}_{i\_SNV\_454}$.

Comparing the RVIs of the selected parameters, *QD* (quality by depth) features the greatest relative variable importance in case of ${\hat{\eta }}_{i\_SNV\_454}$. Regarding ${\hat{\eta }}_{i\_SNV\_IonT}$ *HP* has the greatest RVI value. In case of ${\hat{\eta }}_{i\_SNV\_Illumina}$ *Cov*_*vcf* has the greatest importance according to RVI.

Similar to the GLMs separating true from false positive SNVs, the values of the AIC were also comparable when considering indels (454: 15.45; Ion Torrent: 21.95; Illumina NextSeq: 17.51), indicating a similar performance of all models.

S7 Fig shows that the models successfully assigned a high probability to the little number of true positive indels, while they assigned a low probability to the majority of the false positive indels. Thresholds (*p*_*Indel*_454_ = 0.08, *p*_*Indel*_*IonT*_ = 0.07 and *p*_*Indel*_*Illumina*_ = 0.10) were chosen to retain the original sensitivity and to maximally reduce the number of false positive indels. The last column in Table 8 show the change in PPV if the estimated GLMs are applied. For all platforms PPV is considerably improved: *PPV*_454_ = 0.43, *PPV*_*IonT*_ = 0.64 and *PPV*_*Illumina*_ = 0.86.

Considering indel calling in the training subset after applying the estimated GLMs, Ion Torrent performed equally well compared to 454 and Illumina NextSeq. Ion Torrent data showed the highest sensitivity and a comparable number of false positive calls regarding the other platforms.

To test our approach, the independent test subset was considered. Altogether, only one to three indels were present in the data, which made analysis of sensitivity difficult. Notably, one indel (UPN019: chr20:31,024,457 insA) was not called using the Illumina NextSeq data. In contrast, the indel, resp. the indels were called analyzing the Ion Torrent- and 454 data. Regarding PPV, Illumina NextSeq performed best, while Ion Torrent performed worst—without applying our estimated GLMs.

On the basis of our optimized analysis pipeline (with GLM), Illumina NextSeq and 454 performed best with respect to PPV and Ion Torrent performed worst. Yet, the difference was so small that it seems likely to be due to random effects. An increase in PPV in case of Ion Torrent data from 0.01 to 0.25 was observed. Furthermore, our optimized pipeline successfully identified the true positive indel in case of the Ion Torrent- and Illumina NextSeq data as such, which is why sensitivity was not harmed. However, only one of the three true positive indels in the 454 data was identified by our model approach.

For indel calling it seems that our optimized analysis pipeline leads to better results with respect to PPV in case of Ion Torrent- and Illumina NextSeq data. However, as only one true positive indel could be analyzed, this observation may be due to random effects.

## Discussion

NGS techniques provide an attractive alternative compared to Sanger sequencing with regards to turnaround time and cost of sequencing. However, false positive calls of SNVs and especially indels are a known problem, endangering the use of NGS in clinical routine. If any approach would require confirmation of the many variants called in NGS data with the help of Sanger sequencing, there remains little advantage of the new technology. Consequently, there is a necessity for identifying the platform and analysis pipeline, that perform best in calling variants with equally high sensitivity and precision.

To investigate different NGS platforms, we consider three common sequencers: Roche 454, Ion Torrent PGM and Illumina NextSeq. To investigate different analysis pipelines, we apply a standard pipeline using GATK for variant calling and an optimized, individually calibrated pipeline for every platform and type of variant.

For investigating platform- and variant-specific characteristics, we consider a wide set of different parameters characterizing both SNVs and indels. This set includes standard parameters determined by GATK as well as additional parameters determined by us (see Table 1). We assume that true mutations differ from false positive calls with respect to certain characteristics. By automatically selecting and weighting those characteristics that show the greatest effect in separating true from false positive calls, automatic distinction should be improved.

To optimize variant calling, we consider all parameters and search for those that have the greatest effect on separating true from false positive calls when using a GLM. As our previously performed analysis of the parameters indicated, not only SNVs and indels, but also different NGS technologies are characterized by different flaws. A set of parameters that allow for a perfect distinction between true- and false positive calls in all analyzed scenarios cannot be defined. Therefore, we chose to estimate one GLM per mutation type and per platform. As all models differ considerably from each other (see Eqs (1)–(6)), this assumption appears to be correct.

Instead of one threshold per parameter, only one threshold per model has to be defined. The way a concrete threshold is defined does not only depend on the data, but also on the use case in which the model approach will be applied. With respect to the cost-benefit ratio, we consider it as more acceptable to deal with an additional false positive call—that could be filtered by e.g. expert manual inspection –, than to accidentally exclude a true positive mutation.

### Standard and optimized SNV calling

Taking the standard SNV calling results into account, the analyzed data of the three platforms appear to be equally good regarding sensitivity (see Table 3), although no platform succeeds in calling all SNVs present in the analyzed datasets. With respect to PPV, data indicates a minor advantage in favor of 454 and Illumina NextSeq.

RVI analysis of the 18 parameters characterizing SNVs indicates that their ability to separate true from false positive calls depends on the considered platform. In the context of 454 data, *BQ*_*vcf* (base quality) as well as *Q* (quality) appear to be especially important, while an almost even distribution of variable importance can be observed in case of Ion Torrent data. With respect to Illumina data, *Q* and *SB*_*vcf* (strand bias) feature the highest RIV values by far. Thus, it seems necessary to determine individual optimization approaches for every sequencing platform.

Applying our optimized analysis pipeline leads to a general improvement in the variant calling results (see Table 5). An increase in PPV is observed, while sensitivity is not harmed. The individual GLMs successfully identify all true positive calls and a majority of false positive calls. The results support our initial assumption that true mutations differ from false positive calls in a way that GLMs improve automatic distinction. Based on the optimized results, all sequencing platforms appear to perform equally well concerning sensitivity and PPV.

Analysis of the parameters included in the GLMs shows that *Q* is indeed part of the 454- and the Illumina model. This observation is in line with its previously observed high RVI. *VP*_*vcf* (variant position) is part of both the 454- and the Ion Torrent model. This parameter’s great importance was not expected based on our RVI analysis. Neither parameters characterizing strand bias, nor base quality are part of any of the determined models. Against the background that GATK recommends platform-independent filtration of SNV calls by *QD* (quality by depth)—which we disabled in favor of sensitivity—it appears especially striking that this parameter is not part of any GLM either.

### Standard and optimized indel calling

With regards to the calling of indels, the use of NGS platforms—especially 454 and Ion Torrent—is regarded as problematic due to a high number of false positive calls, especially in the context of homopolymers [5, 7–9, 26]. Therefore, poor results concerning the number of false positive calls were expected in case of these two techniques. For Illumina the lowest number of false positive indels was expected [3] as this sequencing technique relies on a basewise determination of the sequence and the four types of nucleotides are differently labeled.

Standard indel calling results (see Table 6) clearly show the same problem in the samples we analyzed. Both 454 and Ion Torrent sequencing data feature a high number of false positive calls, which is not acceptable in a clinical setting. The sensitivity in case of Ion Torrent sequencing data is high. This is remarkable considering the—in general—lower coverages of Ion Torrent compared to Illumina. Considerably less false positive calls are observed in Illumina NextSeq data.

RVI analysis of the 22 parameters characterizing indels does not only show that their ability to separate true from false positive calls is platform-dependent. The results furthermore reflect considerable differences between SNV- and indel calling. *QD* (quality by depth) and *HP* (homopolymer length) feature outstanding RVIs in case of 454 data. Regarding Ion Torrent data, *SOR* (strand bias) and *HP* have the greatest RVI values. However, regarding Illumina NextSeq data, *DP* (depth) and *DevGT* (indel width), a parameter we developed, showed the greatest importance according to RVI. These results further underline the necessity for individual optimization approaches—for different types of sequencing data as well as for different types of variants.

The application of our optimized pipeline results in a marked decrease in the number of false positive calls. The results support our assumption that true mutations feature different characteristics compared to false positive calls, which is why our GLM approach is successful. All sequencing platforms perform almost equally well when considering optimized indel calling. Only two true positive indels are not identified by our estimated GLM, in case of 454 data. One is a one base pair deletion, which is located in a homopolymer stretch of four T’s and may therefore be difficult to identify by 454 sequencing.

Using the standard analysis pipeline, Ion Torrent appeared to be inferior to the other sequencing techniques, due to the high number of false positive calls. However, using our optimized pipeline, it may prove to be a viable alternative due to highest sensitivity and an improved PPV.

Analysis of the parameters included in the GLMs is in line with the results of our RVI analysis. A heterogeneous set of parameters contributes to separating true- and false positive indel calls in case of the different platforms. As expected, *QD* and *HP* are part of the 454 GLM, while *SOR* and *HP* are part of the Ion Torrent GLM. Interestingly, neither *DP* nor *DevGT* is part of the Illumina model. Instead, *HP*_*AT* is included, but with a positive regression parameter. The probability for an indel call being a true positive thus increases if it is located within an A- or T homopolymer.

Different from the GLMs in case of the optimized SNV calling pipeline, no parameter characterizing the variant position is part of any of the determined models. Furthermore, the intuitively important variance in indel width is not part of any model either. Although GATK recommends filtration of the raw output on the basis of *QD*, it only proved to be relevant for the 454 model.

### Limitations and possible sources of bias

The models we present as a result of our case study are based on a relatively small dataset. We focussed on mutations in a subset of 19 genes that are known to be recurrently mutated in MDS. It would be interesting to explore, whether our approach also works on a thoroughly different, considerably bigger target region. We therefore analyzed sequencing data from three freely available datasets: simulated tumor samples #1, #2 and #3 from the ICGC-TCGA DREAM Mutation Calling challenge [27]. As these are simulated datasets, we can be sure about biological truth. We selected a target region of 1 million base pairs (chr1:186,000,001-187,000,000). Similar to our case study, we use a randomly selected subset of data to train our GLM (samples #1 and #3) and test it on an independent dataset (sample #2). The results can be found in S9 Appendix. Although data show considerable differences compared to our case study, we observe that application of our GLM-based optimization pipeline is successful. Regarding the training set, 154 out of 204 false positive SNV calls (75%) are identified by our GLM. In case of the independent test set, 131 out of 182 false positive SNV calls (72%) are successfully identified by our GLM, while no true positive call is mistaken for a false positive. We therefore assume that the GLM-based optimization approach we present is not restricted to the small target region we analyzed in case of our case study, but it also works on thoroughly different data and a considerably bigger target region.

All calls regarding our case study were categorized as true or false positive by two independent experts. Categorization of only a subset of calls was confirmed by Sanger sequencing. Thus, it is possible that some calls were actually misclassified. However, we were facing a total of 944 calls, i.e. on average more than 47 calls per patient. The high number of calls, but also their low allelic frequency in many cases makes it impossible to validate every call by Sanger sequencing. S8 Fig shows two examples of typical false positive calls that clearly differ from true mutations. A high number of calls is similar to these two examples. Thus, it seems apt to assume that a majority of false positive calls could be identified by manual inspection. For all calls that could not clearly be identified, we considered re-sequencing data, as every sample was sequenced at least twice on a different or the same platform. As we additionally performed Sanger sequencing on a subset of calls, it seems apt to assume that a vast majority of true- and false positive calls was classified correctly.

The fact that considerably shorter reads are analyzed in case of Illumina NextSeq data, may bias variant calling. Shorter reads require a target design with more primers located within the coding regions of larger exons, e.g. ASXL1, exon 13. We use the available primer sequences (TruSight Myeloid Sequencing Panel, see S1 Appendix for details) to determine the primer locations. Subsequently, we use Fisher’s Exact Test to investigate whether the location of an SNV within a primer increases the probability of not detecting this variant in the Illumina NextSeq data. We receive a p value of *p*_*SNV*_ = 0.0029 (one sided test). We adjust *α* = 0.05 using Bonferroni correction (*α* = 0.05/4 = 0.0125). Nevertheless, the result is significant. Thus, it is likely that some of the SNV calls missed by Illumina NextSeq are due to their location within a primer.

The 454 and Ion Torrent samples are completely bidirectionally sequenced, whereas sequencing Illumina NextSeq is mainly unidirectional. Therefore, we also use Fisher’s Exact Test to investigate whether the location of an SNV in an unidirectionally sequenced region increases the probability of not detecting this variant. We receive a p value of *p*_*SNV*_ = 0.9666 (one sided test). Considering the adjusted *α* = 0.0125, a significant result may not be obtained. Thus, it is not likely that this characteristic of Illumina NextSeq data has a negative influence on variant calling in case of SNVs.

Analogous to the calling of SNVs, it is investigated whether the indel calls missed in case of Illumina NextSeq data, may be due to their location in regions where primers align to the coding region of genes. Fisher’s Exact Test leads to the p value *p*_*Indel*_ = 0.1206. Thus, it is unlikely that a dependency of the two variables exists in the case of indels. This means, that the location of primers within coding regions of genes is not likely to prevent an indel from being called in case of Illumina NextSeq data.

In addition, we also test whether the location of an indel in a unidirectionally sequenced region may be responsible for not calling this variant. Fisher’s Exact Test leads to the p value *p*_*Indel*_ = 0.7920. Thus, it is unlikely that the location of an indel in a unidirectionally sequenced region prevents it from being called in Illumina NextSeq data.

We had a detailed look at all mutations and polymorphisms that were present but not called (false negatives). In all cases the variants were visible in the raw alignment data, although some with frequencies as low as 0.03 –. To an on average lesser extent they were also visible in the re-aligned data. Some missed calls were detected by a commercial second analysis approach (see section S8 Appendix). However, they were never called by our analysis software GATK. Analysis of the missed calls does not indicate existence of any platform specific bias (see S1 File).

### Comparison to other optimization approaches

To estimate our individually calibrated GLMs, we use forward selection based on AIC. It appears striking that the application of this parameter selection method did not always lead to the inclusion of those parameters that feature the greatest RVI values. It may thus be argued that our models are not best in separating true from false positive calls. We investigated the effect of an alternative parameter selection method by always including the parameter with the highest RVI (see S10 Table) into our GLMs. However, in no case this method led to an improvement of our models.

It may furthermore be argued that our optimized analysis pipeline using GLMs is a time-consuming and expensive approach. It is not advisable to directly use any of our estimated models for a different set of data generated on the same platforms. The models we estimated are individually calibrated. Not only for the mutation type and the sequencing platform, but also for the laboratory conditions, library preparation, sequencing and the way raw data are generated.

Application of our GLM approach to new data will always require definition of a training set and analysis of this subset according to the pipeline displayed in Fig 1. This also includes validation—by additional experiments or expert-based review—of the variants that have originally been called. Due to the many false positives in the case of Ion Torrent data this step can be problematic. However, only then it will be possible to estimate a GLM that is apt to successfully distinguish true from false positive calls in the given new data and all subsequent data generated according to the same protocol and in the same lab.

It would of course be easier to identify one or two parameters, to set invariable thresholds for these parameters and to filter the calls based on these thresholds. No time consuming validation of the variants would be necessary in this case. However, the laboratory conditions as well as the analysis pipeline have a considerable effect on the results. [9] report an improved straightforward approach for filtering false positive indels from Ion Torrent data when using GATK for variant calling (QD = 2.5 and VARW = 0). We applied this approach to our Ion Torrent training subset. Unfortunately, 71% of the true positives get filtered out. 57% of all true positives feature a value of *QD* < 2.5. Furthermore, 14% of the true positives feature a value of *VARW* ≠ 0. Altogether, 26% of the indels called in the first place get reported as true positives, although they are known to be false positive calls. This stresses the fact that individual models are necessary and a naive filtration on the basis of one or two parameters with invariable thresholds is not advisable.

A thoroughly different approach would be to sequence samples twice—on the same platform or on different platforms. Apart from increasing turnaround time and cost of sequencing, this approach is likely to lead to worse results compared to our model approach. Sequencing platforms are characterized by systematic errors, especially in the case of indel calling (see Table 6, re-sequencing subsets). Therefore, sequencing the same sample twice on the same platform and combining the variant calling results by looking at the overlapping calls can only improve PPV to a limited extent. Furthermore, we observe that true mutations are sometimes missed if a sample gets re-sequenced on the same platform. If applied to a huge dataset, this approach is thus likely to lead to a decrease in sensitivity.

Sequencing the same sample twice on different platforms and considering the overlapping calls is likely to lead to a considerable increase of the PPV (see S1 File). However, the analysis of our comparison subset indicates that this approach is likely to lead to many false negatives and thus to a decrease in sensitivity as well, which is not acceptable in a medical use case.

Optimizing the analysis of NGS data by estimating and applying individual GLMs thus appears to be the most efficient and successful approach for excluding the majority of false positive calls in an automated pipeline. It provides a feasible approach for handling large datasets, e.g. in a trial, for which it is impossible to check all variants manually when using standard analysis.

### Perspective

Despite all possible sources of bias, limitations and alternative filtering strategies, our results appear to be relevant for use of NGS in clinical practice. By using a standard analysis pipeline, none of the considered platforms is able to call all true positive SNVs and indels. For SNVs sensitivity ranges between 0.88 (Illumina NextSeq) and 0.91 (454 and Ion Torrent), while PPV ranges between 0.75 (Ion Torrent) and 0.91 (454 and Illumina NextSeq). For indel calling results sensitivity ranges between 0.75 (454) and 1.00 (Ion Torrent), while PPV ranges between 0.01 (Ion Torrent) and 0.26 (Illumina NextSeq). Our optimized analysis pipeline is able to increase PPV to a considerable extent. Nevertheless, even a sophisticated approach like our filtration on the basis of individually calibrated GLMs is not able to identify all false positive calls and some true positive calls are still missed. If more samples or larger target regions were analyzed, it may be possible to attain more generalizable GLMs and thus more generalizable optimized pipelines.

Altogether, the results of any variant calling pipeline should always be viewed with criticism. Even sophisticated analysis pipelines cannot guarantee that all true variants are called and all false positives are filtered out. Human inspection of the called variants and hotspot mutations that were not called remains necessary.

We suggest to use the optimized pipeline approach together with defining two thresholds instead of one. A majority of false positive calls feature an estimated probability based on the GLMs, which is close to zero. Therefore, a threshold close to zero should be chosen to exclude false positive calls. As a majority of true positive calls feature an estimated probability close to one, a second threshold close to one should be chosen to identify true positive calls. Everything in between these two thresholds could be validated by Sanger sequencing or manual inspection.

## Conclusion

Different from standard analysis pipelines, we present a workflow that provides an effective technique for separating most true from false positive SNVs and indels in the case of Roche 454, Ion Torrent PGM and Illumina NextSeq sequencing data. We thus describe an approach that enables the user to handle even large NGS datasets. Furthermore, our approach is not limited to the three sequencing techniques we analyzed, as the same approach may easily be applied to sequencing data resulting from other platforms. However, our findings also indicate that individual calibration of our workflow is indispensable.

## Supporting information

S1 Appendix Sequencing information. (PDF)

S2 Appendix Read alignment information. (PDF)

S3 Appendix Variant calling and annotation. (PDF)

S4 Appendix Filtration information. (PDF)

S5 Appendix Parameter determination. (PDF)

S6 Appendix Validation information. (PDF)

S7 Appendix Generalized linear model and threshold. (PDF)

S8 Appendix Data analysis—second approach. (PDF)

S9 Appendix TCGA sample analysis. (PDF)

S1 File File providing detailed information on the called- and missed variants. Called SNVs using a standard pipeline and the optimized pipeline, called indels using a standard pipeline and the optimized pipeline, detailed analysis on variants in Illumina NextSeq data and information on missed calls.
(XLS)

S2 File File containing the called SNVs and information on their determined parameters in the TCGA training subset. The file can directly be used as input for the R script determining the best GLM separating true from false positive SNV calls unsing forward selection based on AIC.
(TXT)

S3 File File containing the called SNVs and information on their determined parameters in the TCGA test subset. The file can directly be used to test the GLM, which was estimated on the basis of the training subset.
(TXT)

S1 Script R script determining the 18 parameters characterizing SNVs and 22 parameters characterizing indels called by GATK. (R)

S2 Script R script calculating the relative variable importance (RVI) and determining information for normalization. (R)

S3 Script R script determining the best GLM separating true from false positive SNV calls using forward selection based on AIC. (R)

S4 Script R script determining the best GLM separating true from false positive indel calls using forward selection based on AIC. (R)

S1 Table List of the genes, exons and their ENSEMBL transcript IDs that were targeted by Roche 454, Ion Torrent PGM and Illumina NextSeq. (PDF)

S2 Table Base pairs (bp) in the target region, in exons in the target region and number of genes covered by 454, Ion Torrent and Illumina. (PDF)

S3 Table Alignment statistics for the 454 data aligned with BWA mem. (PDF)

S4 Table Alignment statistics for the Ion Torrent data aligned with TMAP. (PDF)

S5 Table Alignment statistics for the Illumina NextSeq data aligned with BWA mem. (PDF)

S6 Table Number of models containing a parameter and normalized relative variable importance (RVI) for all parameters characterizing SNVs, considering 454, Ion Torrent and Illumina NextSeq sequencing data. (PDF)

S7 TableAkaike’s Information Criterion (AIC), estimates of the regression parameters and their standard error for the linear predictors ${\hat{\eta }}_{i\_SNV\_454}$, ${\hat{\eta }}_{i\_SNV\_IonT}$ and ${\hat{\eta }}_{i\_SNV\_Illumina}$.(PDF)

S8 Table Number of models containing a parameter and normalized relative variable importance (RVI) for all parameters characterizing indels, considering 454, Ion Torrent and Illumina NextSeq sequencing data. (PDF)

S9 TableAkaike’s Information Criterion (AIC), estimates of the regression parameters and their standard error for the linear predictors ${\hat{\eta }}_{i\_Indel\_454}$, ${\hat{\eta }}_{i\_Indel\_IonT}$ and ${\hat{\eta }}_{i\_Indel\_Illumina}$.(PDF)

S10 Table Development of the AIC in case of an alternative parameter selection method based on RVI. (PDF)

S1 Fig Number of samples in the comparison data set with 0x coverage of the genes in the intersecting target region in the case of 454 (black), Ion Torrent (red) and Illumina (green). (TIF)

S2 Fig Median coverage of the genes in the intersecting target region in the case of 454 (black), Ion Torrent (red) and Illumina (green) considering the re-sequencing data set. (TIF)

S3 Fig Number of samples in the re-sequencing data set with 0x coverage of the genes in the intersecting target region in the case of 454 (black), Ion Torrent (red) and Illumina (green). (TIF)

S4 Fig Median coverage of the bases of the intersecting target region in the case of 454 (black), Ion Torrent (red) and Illumina (green) considering the test data set. (TIF)

S5 Fig Number of samples in the test data set with 0x coverage of the genes in the intersecting target region in the case of 454 (black), Ion Torrent (red) and Illumina (green). (TIF)

S6 FigRelation between the estimated probability ${\hat{p}}_{i}$ and the actual probability *p*_*i*_ for an SNV being a true positive.(A) 454 (B) Ion Torrent (C) Illumina; Black circles: training data set; grey diamonds: test data set. Thresholds are displayed as dashed lines (*p*_*SNV*_454_ = 0.28, *p*_*SNV*_*IonT*_ = 0.04 and *p*_*SNV*_*Illumina*_ = 0.07).(TIF)

S7 FigRelation between the estimated probability ${\hat{p}}_{i}$ and the actual probability *p*_*i*_ for an SNV being a true positive.(A) 454 (B) Ion Torrent (C) Illumina; Black circles: training data set; grey diamonds: test data set. Thresholds are displayed as dashed lines (*p*_*Indel*_454_ = 0.08, *p*_*Indel*_*IonT*_ = 0.07 and *p*_*Indel*_*Illumina*_ = 0.01).(TIF)

S8 Fig Typical false positive calls in NGS data. (A) False positive call chr7:148,543,693 TAAAA>T in sample UPN02, Ion Torrent, set2; observed variation in the call (insertion of two A’s up to deletion of five A’s) is strong evidence for a false positive. (B) False positive call chr20:31023122 TG>T in sample UPN007, Ion Torrent, set2; observed strand bias is strong evidence for a false positive.
(TIF)
