# Supplementary material for: GLM-based optimization of NGS data analysis: A case study of Roche 454, Ion Torrent PGM and Illumina NextSeq sequencing data
Source: PLoS One. 2017 Feb 21;12(2):e0171983. doi: 10.1371/journal.pone.0171983 (PMC5319672; doi:10.1371/journal.pone.0171983)
Supplement: S1 Appendix — (PDF) [file pone.0171983.s001.pdf]

Sequencing information

454 Sequencing

Sequencing was performed using Roche 454 technology, largely as described ([1]). Amplicons covering all relevant regions in 20 genes frequently affected in MDS were designed. Pools were prepared for the emulsion PCR (emPCR) step. First, the pools were diluted to a concentration of 4x106 molecules per μl. Secondly, the libraries were processed using the GS FLX Titanium Series Lib-A SV method (Roche Applied Science). Forward (A beads) and reverse (B beads) reactions were carried out using 2,000,000 beads per emulsion oil tube. The copy per bead ratio used was 2.1. The amplification reaction, breaking of the emulsions and enrichment of beads carrying amplified DNA was performed using the workflow as recommended by the manufacturer. However, A beads and B beads were separately processed during breaking and enrichment processes. Finally, samples were loaded on an 8-lane PicoTiterPlate (PTP) on the Genome Sequencer FLX System instrument (Roche Applied Science). All data were generated using the GS FLX Sequencer Instrument software version 2.3. Image processing and amplicon pipeline analysis was performed using default settings of the GS RunBrowser software version 2.3 (Roche Applied Science). Sequence alignment and variant detection was performed using the GS Amplicon Variant Analyzer software version 2.3 (Roche Applied Science). The results were further processed and visualized in R/Bioconductor using the package R453Plus1Toolbox (version 1.0.1).

Ion Torrent Sequencing

Amplicons covering relevant regions of the genes of interest were sequenced using Ion Torrent semiconductor technology, as described[2]. PCRs were performed using conventional sequencing primers in a fully automated robotic work flow. PCR amplicons were subsequently pooled and sheared to 200-300 bp. Library preparation was performed in an automated fashion on a MicroLab Starlet Replicator Robot (Hamilton) by using the Ion Plus fragment library kit in combination with the Ion XpressTM barcode adapters 1-96 kit (both Life Technologies). Emulsion PCRs were performed on an Ion OneTouch system (Ion OT2 instrument, Life Technologies) using the Ion PGM Template OT2 200 kit. Enrichment of template-positive Ion sphere particles (ISPs) was performed on a OneTouch ES system (Life Technologies). The percentage of template-positive ISPs was measured with use of the Ion Sphere Quality Control kit (Life Technologies) and a Qubit 2.0 Fluorometer (Invitrogen). Subsequently, ISPs coated with template were loaded on Ion 318TM sequencing chips (Life Technologies). The chips were sequenced on the PGM, using the Ion PGM sequencing 200 kit version 2.

Illumina NextSeq Sequencing

The genomic library was prepared using 50 ng of DNA template and TruSight DNA Amplicon Sequencing Panel Library Prep Kit (Illumina, San Diego, CA) according to the manufacturer's protocol. Briefly, library preparation involved hybridization of the probe mixture to genomic DNA and areas of interest were

captured by extension and ligation. In addition to areas complimentary to genomic DNA, each of the probes has a common sequence, which is used to PCR-amplify the captured sequences in a subsequent step. This PCR step also adds sequencing adapters (for binding of sequencing primers) and short stretches of identifier sequences or barcodes on the 3' and 5' ends of the amplicons to be used as unique sample identifiers, thus facilitating multiplexed sequencing of the samples. The library generated using the TruSight Myeloid Sequencing Panel was purified using AMPure magnetic beads (Agentcourt, Brea, CA) according to the manufacturer's protocol. From each library, equal quantities of the DNA were eluted using Library normalization beads (TruSight kit) following the manufacturer's instructions and equal volumes were mixed. This ensures similar representation of the library from each sample during multiplexed sequencing. Paired end sequencing of samples was performed using NextSeq 500 mid output Reagent Kit, V2 (2 x 150 cycles) using the NextSeq 500 sequencer (Illumina). On average, libraries from 32 samples per sequencing run were multiplexed. BCL data produced by the NextSeq 500 instruments were converted into demultiplexed fastq files using bcl2fastq 2.14 (Illumina).

References

[1] Kohlmann A, Klein HU, Weissmann S, Bresolin S, Chaplin T, Cuppens H, et al. (2011) The Interlaboratory Robustness of Next-generation sequencing (IRON) study: a deep sequencing investigation of TET2, CBL and KRAS mutations by an international consortium involving 10 laboratories, *Leukemia*, **25**, 1840-1848.

[2] Rothberg JM, Hinz W, Rearick TM, Schultz J, Mileski W, Davey M, et al. (2011) An integrated semiconductor device enabling non-optical genome sequencing, *Nature*, **475**, 348-352, doi:10.1038/nature10242.
