# Supplementary material for: GLM-based optimization of NGS data analysis: A case study of Roche 454, Ion Torrent PGM and Illumina NextSeq sequencing data
Source: PLoS One. 2017 Feb 21;12(2):e0171983. doi: 10.1371/journal.pone.0171983 (PMC5319672; doi:10.1371/journal.pone.0171983)
Supplement: S2 Appendix — (PDF) [file pone.0171983.s002.pdf]

Read alignment

All reads were aligned to the human reference genome hg19 (GRCh37.66) using the Burrows-Wheeler Aligner[2]. In the case of data originating from the 454 and the Illumina NextSeq sequencer, BWA mem (version 0.7.8) was used. In the case of the Ion Torrent data, TMAP (version 3.0.1, [1], http://github.com/iontorrent/tmap) was used, which is an Ion Torrent specific version of BWA.

References

[1] Homer N, Lyons M, Shah M. (2010) TMAP: the torrent mapping program.

[2] Li H, Durbin R. (2009) Fast and accurate short read alignment with Burrows-Wheeler transform, *Bioinformatics*, **25**, 1754-1760.
