# Supplementary material for: GLM-based optimization of NGS data analysis: A case study of Roche 454, Ion Torrent PGM and Illumina NextSeq sequencing data
Source: PLoS One. 2017 Feb 21;12(2):e0171983. doi: 10.1371/journal.pone.0171983 (PMC5319672; doi:10.1371/journal.pone.0171983)
Supplement: S3 Appendix — (PDF) [file pone.0171983.s003.pdf]

Variant calling and annotation

SNV as well as indel calling was mainly performed using GATK version 3.3-0 (HaplotypeCaller)[2]. GATK version 3.4-46 was used in case of the walker VariantAnnotator, because we assume that there might be a bug in version 3.3-0. As it is recommended for DNAseq data, we defined *stand_call_conf=30* and *stand_emit _conf=10.* In basic compliance with the GATK filtering recommendations, the SNV filter was defined as follows: *QD<0.0*, *FS>60.0* and *MQ<40.0.* The indel filter was defined as follows: *QD<0.0*, *FS>200.0*, *ReadPosRankSum<-20.0.* To increase sensitivity we decided to disable the QD filter by setting it to *QD<0.0* instead of following the recommendations.

Variant annotation was performed using SNPeff (version 3.6c, [1]).

References

[1] Cingolani P, Platts A, Coon M, Nguyen T, Wang L, Land SJ, et al. (2012) A program for annotating and predicting the effects of single nucleotide polymorphisms, SnpEff: SNPs in the genome of Drosophila melanogaster strain w1118; iso-2; iso-3, *Fly*, **6**, 80-92.

[2] DePristo M, Banks E, Poplin R, Garimella K, Maguire J, Hartl C, et al. (2011) A framework for variation discovery and genotyping using next-generation DNA sequencing data, *Nat Genet*, **43**, 491-498.
