# Supplementary material for: GLM-based optimization of NGS data analysis: A case study of Roche 454, Ion Torrent PGM and Illumina NextSeq sequencing data
Source: PLoS One. 2017 Feb 21;12(2):e0171983. doi: 10.1371/journal.pone.0171983 (PMC5319672; doi:10.1371/journal.pone.0171983)
Supplement: S4 Appendix — (PDF) [file pone.0171983.s004.pdf]

Filtration information

As the analysis was focussed on coding regions, intronic mutations were excluded. This was achieved by applying a filter only letting pass those variants that provided an annotation containing at least one of the following effect types: *UTR_5_PRIME, UTR_3_PRIME, FRAME_SHIFT, STOP_GAINED, SPLICE_SITE_REGION, NON_SYNONYMOUS_CODING, SYNONYMOUS_CODING, INTRAGENIC,START_LOST, SYNONYMOUS_START, NON_SYNONYMOUS_START, GENE,TRANSCRIPT, EXON, EXON_DELETED, CODON_CHANGE, CODON_INSERTION, CODON_CHANGE_PLUS_CODON_INSERTION, CODON_- DELETION, CODON_CHANGE_PLUS_CODON_DELETION, SYNONYMOUS_STOP, STOP_LOST, RARE_AMINO_ACID.* These refer to the effect types that may – according to SNPeff – be related to an exon or have an exon ID.

In a subsequent filtering step all silent mutations were excluded (effect type: *SILENT*). This filtering step is performed for both called SNVs and indels, as both may give rise to silent mutations.

Furthermore, all called variants located in the 3'- or 5'UTR were excluded from analysis (effect types: *UTR_5_PRIME* and *UTR_3_PRIME*).

To ensure maximum coverage of regions in which SNVs and indels were called, a depth filter was applied excluding all variants that did not provide a total coverage of at least 20 reads (*DP<20*) at the corresponding base position. The number of reads taken into account in this filtering step also considers reads with a low mapping quality.

In a final filtering step of the raw GATK output all variants that are known polymorphisms were excluded. The information on known polymorphisms is taken from on dbSNP ([1]) build 138, excluding all sites after build 129, which results in a list of known polymorphisms.

References

[1] Sherry ST, Ward MH, Kholodov M, Baker J, Phan L, Smigielski EM, et al. (2001), dbSNP: the NCBI database of genetic variation, *Nucleic Acids Res*, **29**, 308-311.
