# Supplementary material for: GLM-based optimization of NGS data analysis: A case study of Roche 454, Ion Torrent PGM and Illumina NextSeq sequencing data
Source: PLoS One. 2017 Feb 21;12(2):e0171983. doi: 10.1371/journal.pone.0171983 (PMC5319672; doi:10.1371/journal.pone.0171983)
Supplement: S5 Appendix — (PDF) [file pone.0171983.s005.pdf]

Parameter determination

For the R script determining all parameters see S1 Script.

Quality and depth

Quality (Q) and depth (DP), as well as the related parameter quality by depth (QD) are taken from the vcf files generated by GATK.

Coverage

Three different types of coverage are considered: All reads according to the bamout files generated by GATK (Cov_total), all reads containing either the reference or the called alternate allele according to the bamout files generated by GATK (Cov_ref) and all reads containing either the reference or the called alternate allele according to the vcf file (Cov_vcf).

Allele frequency

Three different types of allele frequency are considered: The number of reads containing the alternate allele relative to all reads according to the so called bamout files generated by GATK (AF_total), the number of reads containing the alternate allele relative to all reads containing either the reference or the called alternate allele according to the bamout files generated by GATK (AF_ref) and the number of reads containing the alternate allele relative to all reads containing either the reference or the called alternate allele according to the vcf file (AF_vcf).

Strand bias

When sequencing DNA it is – depending on the target enrichment strategy, library preparation and sequencing set up – expected that both the sense (+) and the antisense (-) strand get sequenced to a roughly equal extent at any location in the genome. In our 'configuration' we chose bidirectional sequencing for the 454- and Ion Torrent set up. Consequently, it is expected that a variant appears in the sense and in the antisense strand to an equal extent as well. A deviation from this expectation is called strand bias.

To investigate whether false positive variant calls are associated with strand bias, three parameters are considered.

	Allele	
	Reference	Alternate
+ strand	a	b
- strand	c	d

Fisher's Exact Test is applied to test for the independence of the allele at a defined locus and the number of sense- and antisense strands at that locus. The number of reads (a, b, c and d) thereby refers to the counts according to the bamout files generated by GATK (SB).

Additionally, GATK applies Fisher's Exact Test on a selection of reads. The result is reported in the vcf file (*SB_vcf*) and considered as well as SOR – the Strand Odds Ratio – which is equally calculated by GATK and reported in the vcf (*SOR_vcf*).

Variant position

The relative position of a called variant within the reads covering the location in question, e.g. a location at the very beginning of reads, can be an indication of a false positive.

A Wilcoxon Rank Sum Test on the relative positioning of a variant is performed considering the bamout files generated by GATK. The test is separately performed for the reads aligned to the sense and those aligned to the antisense reference genome. The test explores whether there is a significant difference in the distribution of the relative position of an alternate allele compared to the reference allele in the reads. The smallest p value of the test for the sense and the test for the antisense strand is considered (*VP*).

Additionally, GATK applies Wilcoxon Rank Sum Test on a selection of reads (no differentiation between sense and antisense strands). The result is reported in the vcf file (*VP_vcf*) and considered in our analysis as well.

Base quality

For every called SNV the mean base quality of the reference and alternate bases is calculated. It is separately calculated for the reads aligned to the sense and those aligned to the antisense strand of the reference genome.

As no base quality is assigned to missing bases, the mean base quality can in this case only be calculated for reads providing the reference bases.

A Wilcoxon Rank Sum Test on the quality of the bases – in case of reads with the detected variant and those without – is performed. The test explores whether the alternate bases do feature a significantly lower quality compared to the reference bases (*BQ*). Yet, as missing bases lack an assigned base quality and only one model is estimated for insertions and deletions, the test can only be performed in case of SNVs.

Additionally, GATK applies a Wilcoxon Rank Sum Test on a selection of reads. The result is reported in the vcf file (*BQ_vcf*) and considered in our analysis as well.

Mapping quality

The mapping quality is considered with the help of two parameters. First, it is directly taken from the vcf files generated by GATK (*MQ*). Second, the result of a Wilcoxon Rank Sum Test that is applied by GATK and equally reported in the vcf files is considered (*MQRank*).

Homopolymer length

In case of called indels, the number of identical bases following a call are counted (HP). The number refers to the reference genome. For deletions the count starts at the position of the first missing base. Regarding inserts the count starts at the position of the first not-inserted base.

To investigate whether there might be a difference between A-,resp. T-stretches, i.e. regions that are more loosely held together due to only two hydrogen bonds between A and T, and C-, resp. G-stretches, i.e. regions that are strongly held together due to three hydrogen bonds between C and G, into account, two additional variables are considered. HP_AT considers the length of a homopolymer in case of an A- or T-stretch. Otherwise the variable is zero. HP_CG considers the length of a homopolymer in case of a C- or G-stretch.

Indel width

In case of a true positive indel that is present as a heterozygous variant, reads with two possible lengths concerning the variant can be expected – containing either a deletion and the reference allele, an insert and the reference allele, deletions of two different lengths, inserts of two different lengths or a deletion and an insert.

Homozygous true positive indels are not expected to show any variation regarding the width of deletions, resp. inserts. Thus, all reads are – compared to the reference – expected to contain an insertion or deletion of the same length.

As the commonly used parameter $VARW$[1, 2] does not consider the called genotype, a new parameter called $DevGT$ (*Dev*iation of the width of gaps and inserts from the called *G*eno*T*ype) is introduced. It is calculated as follows:

$$DevGT = \sum_{i=1}^{m} n_i \cdot min\{|(x_i - y_1))|, |(x_i - y_2))|).$$

m is defined as the total number of different variants in the reads at the position of a called indel (e.g. 4 if reads containing a one base pair insert, the reference sequence, a one base pair deletion and a two base pair deletion may be observed). n_i is the total number of reads featuring variant i. x_i defines the number of bases that are added or lack in the considered reads relative to the reference sequence. In case of reads containing an insert, x_i is positive. x_i is negative as regards reads containing a deletion. $x_i = 0$ if the considered reads resemble to the reference. y_1 and y_2 are defined as those values of x_i that refer to the called genotype. In case of a homozygous variant, $y_1 = y_2$. In case of a heterozygous variant, e.g. reference/one base pair deletion, $y_1 = 0$ and $y_2 = -1$.

References

[1] Yeo ZX, Chan M, Yap YS, Ang P, Rozen SG, Lee ASG. (2012) Improving Indel Detection Specificity of the Ion Torrent PGM Benchtop Sequencer, *PLoS One*, **7**, doi:10.1371/journal.pone.0045798.

[2] Yeo ZX, Wong JCL, Rozen SG, Lee ASG. (2014) Evaluation and optimisation of indel detection workflows for ion torrent sequencing of the BRCA1 and BRCA2 genes, *BMC Genomics*, **15**, doi:10.1186/1471-2164-15-516.
