# Supplementary material for: GLM-based optimization of NGS data analysis: A case study of Roche 454, Ion Torrent PGM and Illumina NextSeq sequencing data
Source: PLoS One. 2017 Feb 21;12(2):e0171983. doi: 10.1371/journal.pone.0171983 (PMC5319672; doi:10.1371/journal.pone.0171983)
Supplement: S6 Appendix — (PDF) [file pone.0171983.s006.pdf]

Validation information

When required we performed Sanger-based sequencing on PCR-amplified genomic DNA fragments to confirm sequence variations identified by next generation sequencing.
