# Supplementary material for: GLM-based optimization of NGS data analysis: A case study of Roche 454, Ion Torrent PGM and Illumina NextSeq sequencing data
Source: PLoS One. 2017 Feb 21;12(2):e0171983. doi: 10.1371/journal.pone.0171983 (PMC5319672; doi:10.1371/journal.pone.0171983)
Supplement: S7 Appendix — (PDF) [file pone.0171983.s007.pdf]

Generalized linear model and threshold

For each sequencing platform two generalized linear models (GLMs) were estimated – one for SNVs and one for indels.

As the GLMs were used to separate true from false positive calls, it is assumed that they return a probability by which a called variant is a true positive. The probability distribution was therefore a binomial distribution for both SNVs and indels.

The linear predictor $\boldsymbol{\eta} = \boldsymbol{X\beta}$ was differently defined, depending on whether SNVs or indels were regarded as well as on the platform. Akaike's information criterion (AIC) was used to find the model that best separates true from false positive indels. The covariates were included in the models by applying a forward selection.

At first, a model containing no covariates, just an intercept is estimated (m_0). Its AIC is used for comparison. Subsequently, all possible models containing one covariate are estimated. The model with the lowest AIC (model m_1 containing covariate c_1) is selected and compared to m_0. If m_1 is better, i.e. has a lower AIC, than m_0 it is selected and the forward selection goes on. Otherwise, forward selection stops and m_0 is selected as the final model.

In the second step of forward selection, all possible models containing two covariates are estimated. These models have to fulfil two conditions: (1) The models must contain covariate c_1. (2) The models must not contain any covariate that correlates with c_1. The model with the lowest AIC (m_2) is selected and compared to m_1. If m_2 is better, i.e. has a lower AIC, than m_1 it is selected and the forward selection goes on. Otherwise, forward selection stops and m_1 is selected as the final model.

Forward selection goes on until no more possible covariates remain or no model with a lower AIC can be found.

As link function $g(p_i)$ a logit link is chosen in all cases:

$$\eta_i = g(p_i) = ln(\frac{p_i}{1-p_i}), \qquad \text{for } i = 1, ..., n_{SNV} \text{ and } i = 1, ..., n_{indel}.$$

The threshold for p_i – the probability by which a called variant i is a true positive – was determined to retain the original sensitivity and to reduce the number of false positive calls to a maximum extent.

For the R script determining the best GLMs separating true from false positive calls, see S3 Script and S4 Script.
