# Supplementary material for: GLM-based optimization of NGS data analysis: A case study of Roche 454, Ion Torrent PGM and Illumina NextSeq sequencing data
Source: PLoS One. 2017 Feb 21;12(2):e0171983. doi: 10.1371/journal.pone.0171983 (PMC5319672; doi:10.1371/journal.pone.0171983)
Supplement: S8 Appendix — (PDF) [file pone.0171983.s008.pdf]

Data analysis – second approach

Sequence alignment and variant detection was performed using the SeqNext module of Sequence Pilot software, version 4.1.2 (JSI medical systems, Germany). Within SeqNext, the sequencing reads were mapped to defined ROIs, and variant calling was performed using user-defined settings. Analysis parameters in combination with selective procedures were used to ensure high coverage and high sensitivity, thus taking specific sequencing technology-based limitations into account (e.g., bases with low base call quality and homopolymer topics). For the detection of variants, filters were set to display sequence variants occurring in more than 10% of reads per amplicon. Mutations within introns (with the exception of mutations affecting splice site) and single nucleotide polymorphisms (SNPs; dbSNP build 131) were discarded.
