# Supplementary material for: GLM-based optimization of NGS data analysis: A case study of Roche 454, Ion Torrent PGM and Illumina NextSeq sequencing data
Source: PLoS One. 2017 Feb 21;12(2):e0171983. doi: 10.1371/journal.pone.0171983 (PMC5319672; doi:10.1371/journal.pone.0171983)
Supplement: S9 Appendix — (PDF) [file pone.0171983.s009.pdf]

TCGA sample analysis

To test our GLM-based optimization approach for NGS data analysis on a thoroughly different dataset, we consider three freely available samples originating from the ICGC-TCGA DREAM Mutation Calling Challenge [1] (download information: https://www.synapse.org/#!Synapse:syn312572/wiki/60898; tumor BAM UUIDs: https://www.synapse.org/#!Synapse:syn2280639; ground truth: https://www.synapse.org/#!Synapse:syn312572/wiki/60874). We analyze simulated tumor samples #1, #2 and #3 according to our optimized variant calling pipeline. All of the simulated tumor samples are WGS samples. We focus our analysis on 1 million base pairs (chr1:186,000,001-187,000,000). Due to the different characteristics of WGS data compared to targeted sequencing data, we do not exclude any mutations because of low coverage (original filter: exclude mutations with coverage<20x). Furthermore, we know that data contain true mutations outside the exon. Therefore, we consider all mutations except for silent mutations (original filter: exclude intronic mutations and mutations in the 3'- or 5'UTR).

The variant calling results concerning SNVs can be found in the following table:

Table 1: *

True- and false positive SNV calls, sensitivity (sens) and PPV considering the TCGA training subset ($n = 2$) and the TCGA test subset ($n = 1$), comparing the standard analysis pipleine (without GLM) and the optimized analysis pipleine (with GLM).

Dataset	SNVs without GLM				SNVs with GLM			
	SNVs	False Positives	Sens	PPV	SNVs	False Positives	Sens	PPV
Training	5	204	1.00	0.02	5	50	1.00	0.09
Test	5	182	1.00	0.03	5	51	1.00	0.09

GATK succeeds in calling all true positive calls in the investigated target region in case of the TCGA training subset. However, we observe poor results regarding PPV. Using the information on the SNVs that were called in the training subset, we estimated a GLM just like in case of the case study. The linear predictor $\hat{n}_{i\_SNV\_TCGA}$ leading to the best results is defined as follows:

$$\hat{n}_{i\_SNV\_TCGA} = 2.98 - 21.55 \cdot x_{i\_SB} + 0.01 \cdot x_{i\_DP} \quad (1)$$

The model features a thoroughly different set of covariates compared to the models we estimated in case of the case study. The AIC is $AIC = 40.32$, the threshold is $p_{SNV\_TCGA} = 0.02$. It is clear that application of our GLM approach leads to a considerable improvement in the variant calling results. Roughly 75% of the false positive calls are identified as such, while no true positive calls are filtered by our estimated model. This is true for the training, but also for the independent test set. We therefore assume that the GLM-based optimization approach we present is not restricted to the small target region we analyzed in case of the case study, but it also works for thoroughly different data and a considerably bigger target region.

References

[1] Ewing AD, Houlahan KE, Hu Y, Ellrott K, Caloian C, Yamaguchi TN, et al. (2015) Combining tumor genome simulation with crowdsourcing to benchmark somatic single-nucleotide-variant detection, *Nat Meth*, **12**, doi:10.1038/nmeth.3407.
