# Supplementary material for: GLM-based optimization of NGS data analysis: A case study of Roche 454, Ion Torrent PGM and Illumina NextSeq sequencing data
Source: PLoS One. 2017 Feb 21;12(2):e0171983. doi: 10.1371/journal.pone.0171983 (PMC5319672; doi:10.1371/journal.pone.0171983)
Supplement: S1 Table — (PDF) [file pone.0171983.s017.pdf]

Table 1: List of the genes, exons and their ENSEMBL [1] transcript IDs that were targeted by Roche 454, Ion Torrent PGM and Illumina NextSeq.

Gene	Exons	TranscriptID
ASXL1	E13	ENST00000375687
CBL	E8, E9	ENST00000264033
DNMT3A	E2-E23	ENST00000264709
ETV6	E1-E8	ENST00000396373
EZH2	E2-E20	ENST00000320356
FLT3	E20	ENST00000241453
IDH1	E4	ENST00000345146
IDH2	E4	ENST00000330062
JAK2	E12, E14	ENST00000381652
KRAS	E2, E3	ENST00000256078
NPM1	E11	ENST00000296930
NRAS	E2, E3	ENST00000369535
RUNX1	E3-E8	ENST00000437180
SF3B1	E13, E14, E16	ENST00000392485
SRSF2	E1	ENST00000392485
TET2	E3-E11	ENST00000380013
TP53	E2-E11	ENST00000269305
U2AF1	E2	ENST00000291552
ZRSR2	E1-E11	ENST00000307771

References

[1] Flicek P, Ridwan Amode M, Barrell D, Beal K, Billis K, Brent S, et al. (2014) Ensembl 2014, *Nucleic Acids Res*, **24**, doi: 10.1093/nar/gkt1196.
