# Supplementary material for: GLM-based optimization of NGS data analysis: A case study of Roche 454, Ion Torrent PGM and Illumina NextSeq sequencing data
Source: PLoS One. 2017 Feb 21;12(2):e0171983. doi: 10.1371/journal.pone.0171983 (PMC5319672; doi:10.1371/journal.pone.0171983)
Supplement: S2 Table — (PDF) [file pone.0171983.s018.pdf]

Table 1: Base pairs (bp) in the target region, in exons in the target region and number of genes covered by 454, Ion Torrent and Illumina.

Sequencer	Target region (total)	Target region (exon)	Genes
454	40,629 bp	24,931 bp	20
Ion Torrent	47,588 bp	25,536 bp	20
Illumina	108,329 bp	71,803 bp	54
Overlap	28,775 bp	22,062 bp	19
