# Supplementary material for: GLM-based optimization of NGS data analysis: A case study of Roche 454, Ion Torrent PGM and Illumina NextSeq sequencing data
Source: PLoS One. 2017 Feb 21;12(2):e0171983. doi: 10.1371/journal.pone.0171983 (PMC5319672; doi:10.1371/journal.pone.0171983)
Supplement: S3 Table — (PDF) [file pone.0171983.s019.pdf]

Table 1: Alignment statistics for the 454 data aligned with BWA mem.

| Sample | UPN001 | UPN002 | UPN003 | UPN004 | UPN005 | UPN006 | UPN007 | UPN008 |
|---|---|---|---|---|---|---|---|---|
| Read length (bp) | 20-700 | 20-721 | 20-904 | 20-793 | 20-719 | 20-824 | 20-588 | 20-554 |
| Total reads | 37,664 | 48,587 | 50,578 | 62,723 | 39,889 | 58,325 | 65,119 | 58,321 |
| Mapped reads | 36,998 | 47,167 | 48,887 | 60,902 | 39,290 | 56,780 | 63,490 | 56,296 |
| Mapped reads | 98.2% | 97.1% | 96.7% | 97.1% | 98.5% | 97.4% | 97.5% | 96.5% |
| Uniquely mapped reads | 36,950 | 47,132 | 48,853 | 60,785 | 39,282 | 56,740 | 63,448 | 56,262 |
| Uniquely mapped reads | 99.9% | 99.9% | 99.9% | 99.8% | 100.0% | 99.9% | 99.9% | 99.9% |
| Reads on target | 35,199 | 42,669 | 45,335 | 55,998 | 37,162 | 51,837 | 60,148 | 52,902 |
| Reads on target | 95.3% | 90.5% | 92.8% | 92.1% | 94.6% | 91.4% | 94.8% | 94.0% |
| Target bases larger 1x | 28,483 | 28,775 | 28,775 | 28,301 | 28,460 | 28,775 | 27,789 | 28,239 |
| Target bases larger 1x | 99.0% | 100.0% | 100.0% | 98.4% | 98.9% | 100.0% | 96.6% | 98.2% |
| Target bases larger 50x | 18,353 | 26,692 | 25,579 | 24,866 | 26,646 | 27,978 | 25,082 | 26,636 |
| Target bases larger 50x | 63.8% | 92.8% | 89.0% | 86.4% | 92.6% | 97.2% | 87.2% | 92.6% |

| Sample | UPN009 set 1 | UPN009 set 2 | UPN010 set 1 | UPN010 set 2 | UPN011 set 1 | UPN011 set 2 | UPN012 set 1 | UPN012 set 2 |
|---|---|---|---|---|---|---|---|---|
| Read length (bp) | 20-843 | 20-697 | 20-801 | 20-950 | 20-868 | 20-549 | 20-781 | 20-761 |
| Total reads | 159,460 | 54,126 | 138,248 | 47,672 | 141,893 | 48,765 | 165,250 | 51,079 |
| Mapped reads | 155,187 | 52,658 | 135,095 | 46,549 | 138,519 | 47,601 | 160,971 | 50,046 |
| Mapped reads | 97.3% | 97.3% | 97.7% | 97.6% | 97.6% | 97.6% | 97.4% | 98.0% |
| Uniquely mapped reads | 155,003 | 52,610 | 134,904 | 46,516 | 138,402 | 47,575 | 160,793 | 49,993 |
| Uniquely mapped reads | 99.9% | 99.9% | 99.9% | 99.9% | 99.9% | 99.9% | 99.9% | 99.9% |
| Reads on target | 73,946 | 49,002 | 64,236 | 44,591 | 68,381 | 45,095 | 79,374 | 47,395 |
| Reads on target | 47.7% | 93.1% | 47.6% | 95.9% | 49.4% | 94.8% | 49.4% | 94.8% |
| Target bases larger 1x | 28,775 | 28,460 | 28,775 | 27,757 | 28,775 | 28,460 | 28,775 | 28,571 |
| Target bases larger 1x | 100.0% | 98.9% | 100.0% | 96.5% | 100.0% | 98.9% | 100.0% | 99.3% |
| Target bases larger 50x | 28,584 | 27,335 | 27,911 | 25,911 | 28,269 | 26,531 | 28,269 | 25,814 |
| Target bases larger 50x | 99.3% | 95.0% | 97.0% | 90.0% | 98.2% | 92.2% | 98.2% | 89.7% |

| Sample | UPN013 set 1 | UPN013 set 2 | UPN019 | UPN020 |
|---|---|---|---|---|
| Read length (bp) | 20-917 | 20-993 | 20-780 | 20-680 |
| Total reads | 163,712 | 36,569 | 49,828 | 76,457 |
| Mapped reads | 159,507 | 35,855 | 48,801 | 74,372 |
| Mapped reads | 97.4% | 98.0% | 97.9% | 97.3% |
| Uniquely mapped reads | 159,357 | 35,838 | 52,610 | 74,335 |
| Uniquely mapped reads | 99.9% | 100.0% | 99.9% | 100.0% |
| Reads on target | 79,387 | 33,543 | 45,999 | 65,616 |
| Reads on target | 49.8% | 93.6% | 94.3% | 88.3% |
| Target bases larger 1x | 28,460 | 28,460 | 28,514 | 28,775 |
| Target bases larger 1x | 98.9% | 98.9% | 99.1% | 100.0% |
| Target bases larger 50x | 28,074 | 27,416 | 28,322 | 28,191 |
| Target bases larger 50x | 97.6% | 95.3% | 98.4% | 98.0% |
