# Supplementary material for: GLM-based optimization of NGS data analysis: A case study of Roche 454, Ion Torrent PGM and Illumina NextSeq sequencing data
Source: PLoS One. 2017 Feb 21;12(2):e0171983. doi: 10.1371/journal.pone.0171983 (PMC5319672; doi:10.1371/journal.pone.0171983)
Supplement: S4 Table — (PDF) [file pone.0171983.s020.pdf]

Table 1: Alignment statistics for the Ion Torrent data aligned with TMAP.

Sample	UPN001 set 1	UPN001 set 2	UPN002 set 1	UPN002 set 2	UPN003 set 1	UPN003 set 2	UPN004 set 1	UPN004 set 2
Read length (bp)	8-322	8-343	8-354	8-336	8-327	25-343	8-352	8-330
Total reads	723,271	731,156	975015	1282301	375,240	680,995	1,536,381	483,779
Mapped reads	701,401	709214	949,543	1240647	355,600	666,797	1,491,351	474,572
Mapped reads	97.0%	93.6%	97.4%	96.8%	94.8%	97.9%	97.1%	98.1%
Uniquely mapped reads	697,375	938,896	946,114	1,230,379	350,190	664,794	1,480,900	472,900
Uniquely mapped reads	99.4%	98.4%	99.6%	99.2%	98.5%	99.7%	99.3%	99.6%
Reads on target	553,816	554,148	821,546	951,400	275,605	529,455	1,117,600	392,590
Reads on target	79.4%	78.6%	86.8%	77.3%	78.7%	79.6%	75.5%	83.0%
Target bases larger 1x	28,775	28775	25,476	28775	28,775	28775	28,775	26,510
Target bases larger 1x	100.0%	100.0%	88.5%	100.0%	100.0%	100.0%	100.0%	92.1%
Target bases larger 50x	28,775	28775	21,109	28480	27,827	28,775	28,775	25,177
Target bases larger 50x	100.0%	100.0%	73.4%	99.0%	96.7%	100.0%	100.0%	87.5%
Sample	UPN005 set 1	UPN005 set 2	UPN006	UPN007	UPN008	UPN009	UPN020	
Read length (bp)	8-336	8-355	8-356	8-326	8-354	8-344	8-322	
Total reads	685,896	781,493	1,666,132	454,784	998,317	546,595	558,860	
Mapped reads	665,699	765,124	1603570	439,239	978,378	516,241	543,763	
Mapped reads	97.1%	97.9%	96.2%	96.6%	98.0%	94.4%	97.3%	
Uniquely mapped reads	662,381	760,969	1,593,994	436,318	973,545	511,949	540,470	
Uniquely mapped reads	99.5%	99.5%	99,4%	99.3%	99.5%	99.2%	99.4%	
Reads on target	523,543	570,432	1,291,147	340,374	765,665	398,322	413,641	
Reads on target	79.0%	75.0%	81.0%	78.0%	78.6%	77.8%	76.5%	
Target bases larger 1x	27,624	28,775	28,775	28,775	28,775	28,592	28,775	
Target bases larger 1x	96.0%	100.0%	100.0%	100.0%	100.0%	99.4%	100.0%	
Target bases larger 50x	27,558	28,702	28,689	28,478	28,775	28,556	28,775	
Target bases larger 50x	95.8%	99.7%	99.7%	99.0%	100.0%	99.2%	100.0%	
