# Supplementary material for: GLM-based optimization of NGS data analysis: A case study of Roche 454, Ion Torrent PGM and Illumina NextSeq sequencing data
Source: PLoS One. 2017 Feb 21;12(2):e0171983. doi: 10.1371/journal.pone.0171983 (PMC5319672; doi:10.1371/journal.pone.0171983)
Supplement: S5 Table — (PDF) [file pone.0171983.s021.pdf]

Table 1: Alignment statistics for the Illumina NextSeq data aligned with BWA mem.

Sample	UPN001	UPN002	UPN003	UPN004	UPN005	UPN006	UPN007
Read length (bp)	30-151	30-151	30-151	30-151	30-151	30-151	30-151
Total reads	9,644,091	11,030,163	9,403,182	9,591,867	12,639,755	3,686,454	13,233,207
Mapped reads	9607613	10986554	9359193	9,551,866	12588918	3672371	13,177,642
Mapped reads	99.6%	99.6%	99.5%	99.6%	99.6%	99.6%	99.6%
Uniquely mapped reads	9577230	10945847	9325440	9,517,955	12,545,243	3,656,702	13,126,776
Uniquely mapped reads	99.7%	99.6%	99.6%	99.6%	99.7%	99.6%	99.6%
Reads on target	4,001,811	3,728,403	3879811	3,356,753	4867466	1,353,052	4,206,983
Reads on target	41.8%	34.1%	41.6%	35.3%	38.8%	37.0%	32.0%
Target bases larger 1x	28775	28775	28775	28,775	28,775	28,775	28,775
Target bases larger 1x	100.0%	100.0%	100.0%	100.0%	100.0%	100.0%	100.0%
Target bases larger 50x	28702	28703	28747	28,703	28,703	28,702	28,703
Target bases larger 50x	99.7%	99.7%	99.9%	99.7%	99.7%	99.7%	99.7%

Sample	UPN008	UPN009	UPN014 set 1	UPN014 set 2	UPN015 set 1	UPN015 set 2	UPN016 set 1
Read length (bp)	30-151	30-151	30-151	30-151	30-151	30-151	30-151
Total reads	9378433	3430992	4454017	3133618	5395630	10497590	3552334
Mapped reads	9339169	3416932	4394118	3120843	5376218	10452740	3479284
Mapped reads	99.6%	99.6%	98.7%	99.6%	99.6%	99.6%	97.9%
Uniquely mapped reads	9299820	3402451	4331918	3107848	5356373	10413135	3424405
Uniquely mapped reads	99.6%	99.6%	98.6%	99.6%	99.6%	99.6%	98.4%
Reads on target	3604703	1338117	1690421	1214057	1868689	3836034	1319305
Reads on target	38.8%	39.3%	39.0%	39.1%	34.9%	36.8%	38.5%
Target bases larger 1x	28775	28775	28775	28775	28702	28775	28775
Target bases larger 1x	100.0%	100.0%	100.0%	100.0%	99.7%	100.0%	100.0%
Target bases larger 50x	28,702	28702	28702	28702	28578	28703	28702
Target bases larger 50x	99.7%	99.7%	99.7%	99.7%	99.3%	99.7%	99.7%

Sample	UPN016 set 2	UPN017 set 1	UPN017 set 2	UPN018 set 1	UPN018 set 2	UPN019
Read length (bp)	30-151	30-151	30-151	30-151	30-151	30-151
Total reads	10582351	8,586,710	11186782	9888890	3917208	6990940
Mapped reads	10543618	8,540,969	11108122	9815912	3904017	6,932,321
Mapped reads	99.6%	99.5%	99.3%	99.3%	99.7%	99.2%
Uniquely mapped reads	10506625	8,511,191	11064241	9767604	3893369	6,887,177
Uniquely mapped reads	99.6%	99.7%	99,6	99.5%	99.7%	99.3%
Reads on target	4115565	3601511	4663208	3246807	1328907	2,251,240
Reads on target	39.2%	43.6%	42.1%	33.2%	34.1%	32.7%
Target bases larger 1x	28775	28775	28775	28775	28775	28,775
Target bases larger 1x	100.0%	100.0%	100.0%	100.0%	100.0%	100.0%
Target bases larger 50x	28704	28608	28679	28489	28702	28702
Target bases larger 50x	99.8%	99.4%	99.7%	99.0%	99.7%	99.7%
