# Supplementary material for: GLM-based optimization of NGS data analysis: A case study of Roche 454, Ion Torrent PGM and Illumina NextSeq sequencing data
Source: PLoS One. 2017 Feb 21;12(2):e0171983. doi: 10.1371/journal.pone.0171983 (PMC5319672; doi:10.1371/journal.pone.0171983)
Supplement: S6 Table — (PDF) [file pone.0171983.s022.pdf]

Table 1: Number of models containing a parameter and normalized relative variable importance (RVI) for all parameters characterizing SNVs, considering 454, Ion Torrent and Illumina NextSeq sequencing data.

Parameter	454		Ion Torrent		Illumina	
	♯models	RVI	♯models	RVI	♯models	RVI
Q	301	**2.04**	837	0.6	142	**4.12**
DP	571	0.58	773	0.61	348	0.29
QD	171	1.73	477	0.81	0	0
Cov_total	348	1.01	694	1	219	**1.15**
Cov_ref	347	1.01	693	1	219	1.14
Cov_vcf	303	0.81	361	0.55	170	0.32
AF_total	238	0.71	468	0.78	0	0
AF_ref	247	0.74	467	0.78	0	0
AF_vcf	361	1	612	0.69	0	0
SB	353	0.38	264	0.17	277	0.44
SB_vcf	445	0.85	75	0.7	142	2.12
SOR	172	0.8	1595	1.26	148	0.56
VP	600	0.67	834	0.63	258	0.41
VP_vcf	202	**0.93**	523	**0.83**	291	0.73
BQ	569	0.84	623	0.73	260	0.66
BQ_vcf	58	2.91	564	0.8	260	1.08
MQ	693	1.07	467	0.76	330	1.20
$MQRank$	185	0.45	1081	0.84	186	0.30
Altogether	1571		2181		847	
