# Supplementary material for: GLM-based optimization of NGS data analysis: A case study of Roche 454, Ion Torrent PGM and Illumina NextSeq sequencing data
Source: PLoS One. 2017 Feb 21;12(2):e0171983. doi: 10.1371/journal.pone.0171983 (PMC5319672; doi:10.1371/journal.pone.0171983)
Supplement: S7 Table — (PDF) [file pone.0171983.s023.pdf]

Table 1: Akaike's Information Criterion (AIC), estimates of the regression parameters and their standard error for the linear predictors $\hat{\eta}_{i\_SNV\_454}$, $\hat{\eta}_{i\_SNV\_IonT}$ and $\hat{\eta}_{i\_SNV\_Illumina}$.

Linear predictor	AIC	Covariate	Estimate	Std. Error
$\hat{\eta}_{i\_SNV\_454}$	10.75	Intercept	-7.08	5.63
		Q	0.02	0.02
		VP_vcf	6.70	5.49
$\hat{\eta}_{i\_SNV\_IonT}$	9.09	Intercept	-4.58	3.80
		VP_vcf	20.62	14.59
$\hat{\eta}_{i\_SNV\_Illumina}$	12.01	Intercept	9.09	5.84
		Q	0.01	0.002
		Cov_total	-0.02	0.01
