# Supplementary material for: GLM-based optimization of NGS data analysis: A case study of Roche 454, Ion Torrent PGM and Illumina NextSeq sequencing data
Source: PLoS One. 2017 Feb 21;12(2):e0171983. doi: 10.1371/journal.pone.0171983 (PMC5319672; doi:10.1371/journal.pone.0171983)
Supplement: S8 Table — (PDF) [file pone.0171983.s024.pdf]

Table 1: Number of models containing a parameter and normalized relative variable importance (RVI) for all parameters characterizing indels, considering 454, Ion Torrent and Illumina NextSeq sequencing data.

Parameter	454		Ion Torrent		Illumina	
	♯models	RVI	♯models	RVI	♯models	RVI
Q	4920	0.54	26414	0.74	11474	**2.09**
DP	5356	0.28	26615	0.46	12752	2.00
QD	1069	**5.68**	13323	2.06	11145	0.42
Cov_total	2670	0.32	16703	**1.02**	11526	0.63
Cov_ref	2672	0.35	16683	0.99	11434	0.49
Cov_vcf	3172	0.44	16671	0.61	9759	**1.39**
AF_total	1682	2.90	16679	0.73	8584	0.95
AF_ref	2929	1.42	16696	0.61	12771	0.58
AF_vcf	3277	0.22	16549	1.70	10629	0.24
SB	3034	0.27	17280	0.00	11382	1.02
SB_vcf	2564	0.12	17263	0.02	11027	0.79
SOR	2322	0.63	15057	**4.42**	11306	0.58
VP	1356	0.19	22173	1.30	12592	1.74
VP_vcf	4875	0.36	22317	0.49	16110	0.34
BQ_vcf	5585	**0.26**	33257	0.41	21903	0.44
MQ	3795	0.35	21131	**1.65**	15988	0.61
$MQRank$	3657	0.39	22828	0.70	13635	0.76
HP	788	**3.16**	15865	**2.59**	10086	1.24
HP_AT	3499	0.10	17239	0.24	10746	**0.76**
HP_CG	3444	0.69	16509	0.90	11834	0.74
$VARW$	1544	0.35	22204	0.81	16456	0.60
$DevGT$	1113	0.74	22284	0.56	11630	1.87
Altogether	11417		66881		45748	
