# Supplementary material for: GLM-based optimization of NGS data analysis: A case study of Roche 454, Ion Torrent PGM and Illumina NextSeq sequencing data
Source: PLoS One. 2017 Feb 21;12(2):e0171983. doi: 10.1371/journal.pone.0171983 (PMC5319672; doi:10.1371/journal.pone.0171983)
Supplement: S9 Table — (PDF) [file pone.0171983.s025.pdf]

Table 1: Akaike's Information Criterion (AIC), estimates of the regression parameters and their standard error for the linear predictors $\hat{\eta}_{i\_Indel\_454}$, $\hat{\eta}_{i\_Indel\_IonT}$ and $\hat{\eta}_{i\_Indel\_Illumina}$.

Linear predictor	AIC	Covariate	Estimate	Std. Error
$\hat{\eta}_{i\_Indel\_454}$	15.45	Intercept	27.06	4755.18
		HP	-31.02	4755.18
		QD	0.58	0.43
		BQ_vcf	16.69	11.18
$\hat{\eta}_{i\_Indel\_IonT}$	21.95	Intercept	-64.76	29.43
		SOR	-4.02	1.93
		HP	-2.93	1.19
		MQ	0.81	0.36
		Cov_total	0.01	0.01
$\hat{\eta}_{i\_Indel\_Illumina}$	17.51	Intercept	-12.09	6.31
		Cov_vcf	0.11	0.06
		Q	-0.01	0.003
		HP_AT	2.51	1.37
