# Supplementary material for: GLM-based optimization of NGS data analysis: A case study of Roche 454, Ion Torrent PGM and Illumina NextSeq sequencing data
Source: PLoS One. 2017 Feb 21;12(2):e0171983. doi: 10.1371/journal.pone.0171983 (PMC5319672; doi:10.1371/journal.pone.0171983)
Supplement: S10 Table — (PDF) [file pone.0171983.s026.pdf]

Table 1: Development of the AIC in case of an alternative parameter selection method based on RVI.

Variant	Sequencer	AIC (GLM)	Included parameter	AIC
SNVs	454	10.75	BA_vcf	15.78
			Q	not converging
	Ion Torrent	9.09	SOR	not converging
	Illumina	12.01	Q	13.21
			SB_vcf	14.39
Indels	454	15.45	QD	33.47
			HP	16.77
			AF_total	not converging
	Ion Torrent	21.95	SOR	44.55
			HP	28.61
			QD	30.28
	Illumina	17.51	Q	47.17
			DP	23.94
			$DevGT$	23.26
			VP	21.67
			Cov_vcf	22.85
