# Supplementary figures and images for: GLM-based optimization of NGS data analysis: A case study of Roche 454, Ion Torrent PGM and Illumina NextSeq sequencing data
Source: PLoS One. 2017 Feb 21;12(2):e0171983. doi: 10.1371/journal.pone.0171983 (PMC5319672; doi:10.1371/journal.pone.0171983)

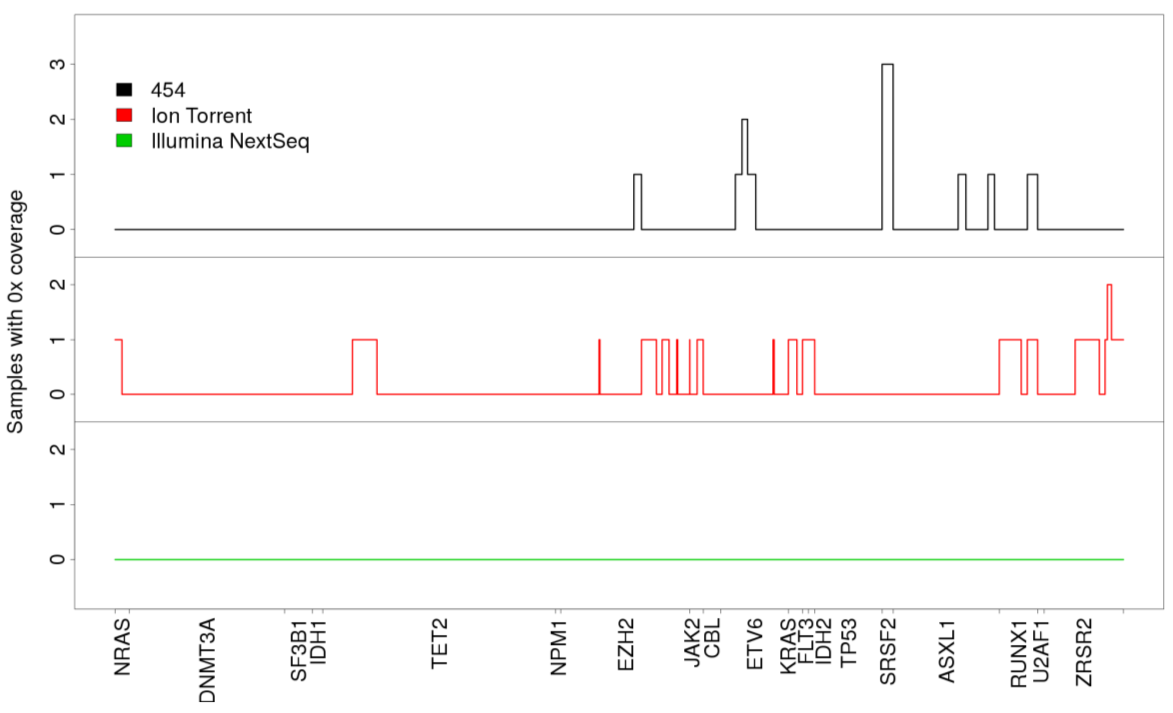

Supplement: S1 Fig — (TIF) [file pone.0171983.s027.tif]

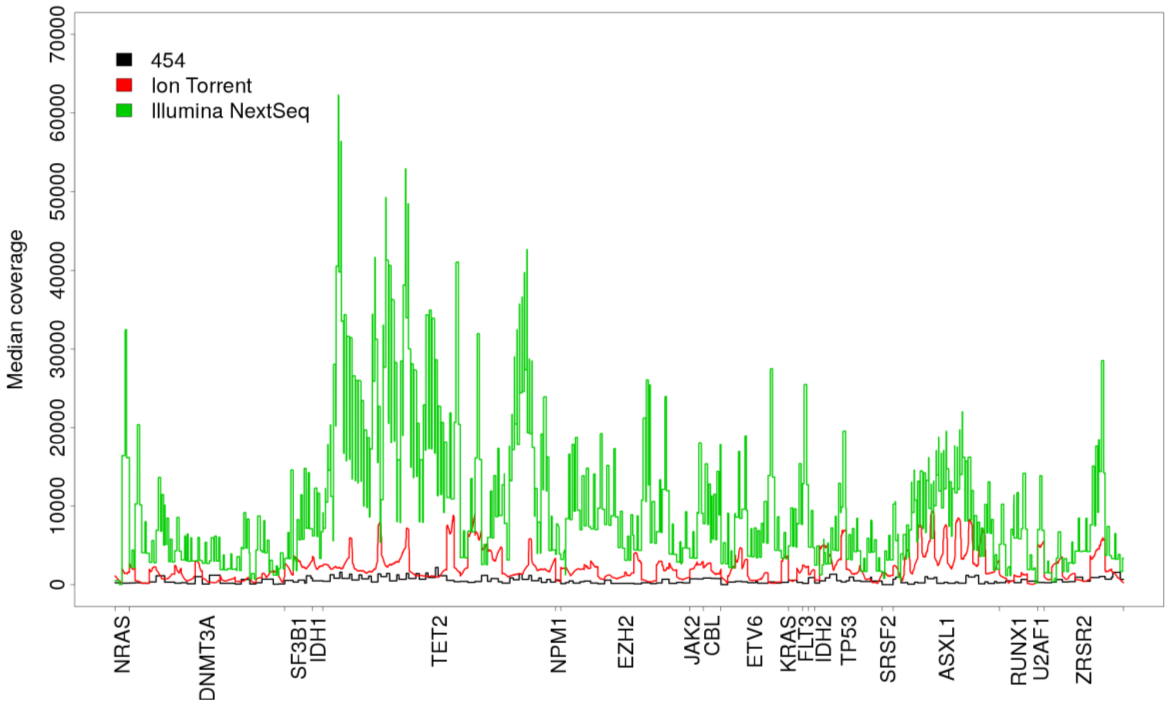

Supplement: S2 Fig — (TIF) [file pone.0171983.s028.tif]

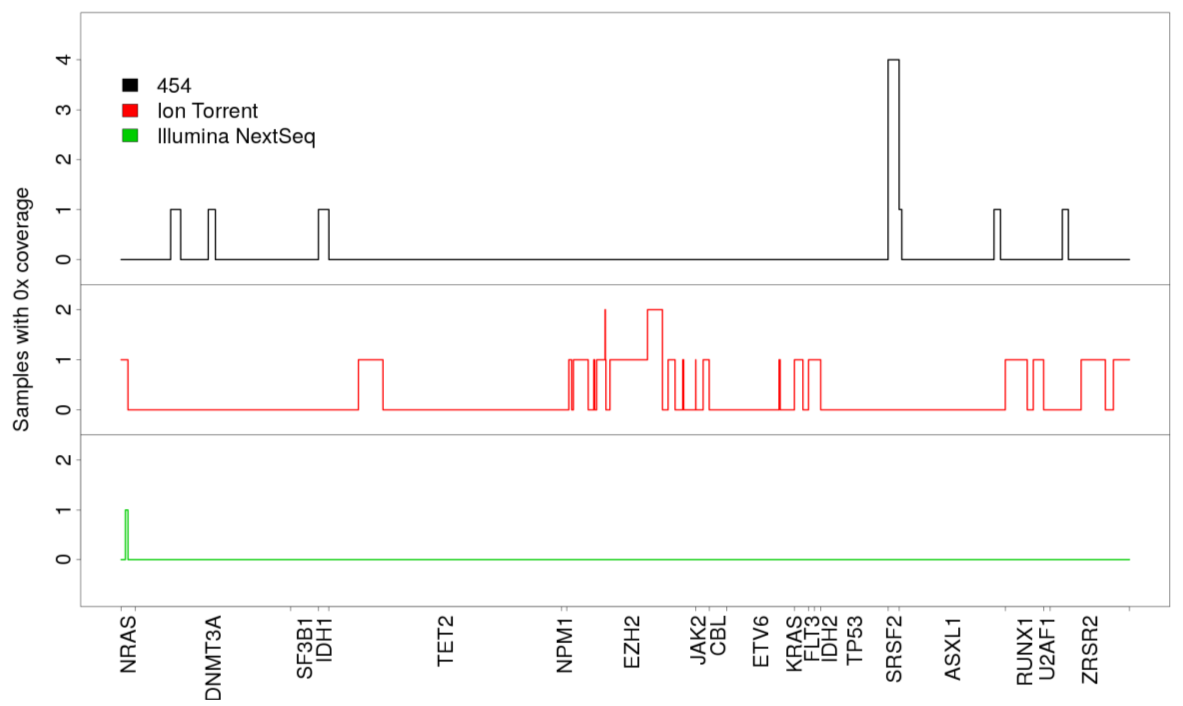

Supplement: S3 Fig — (TIF) [file pone.0171983.s029.tif]

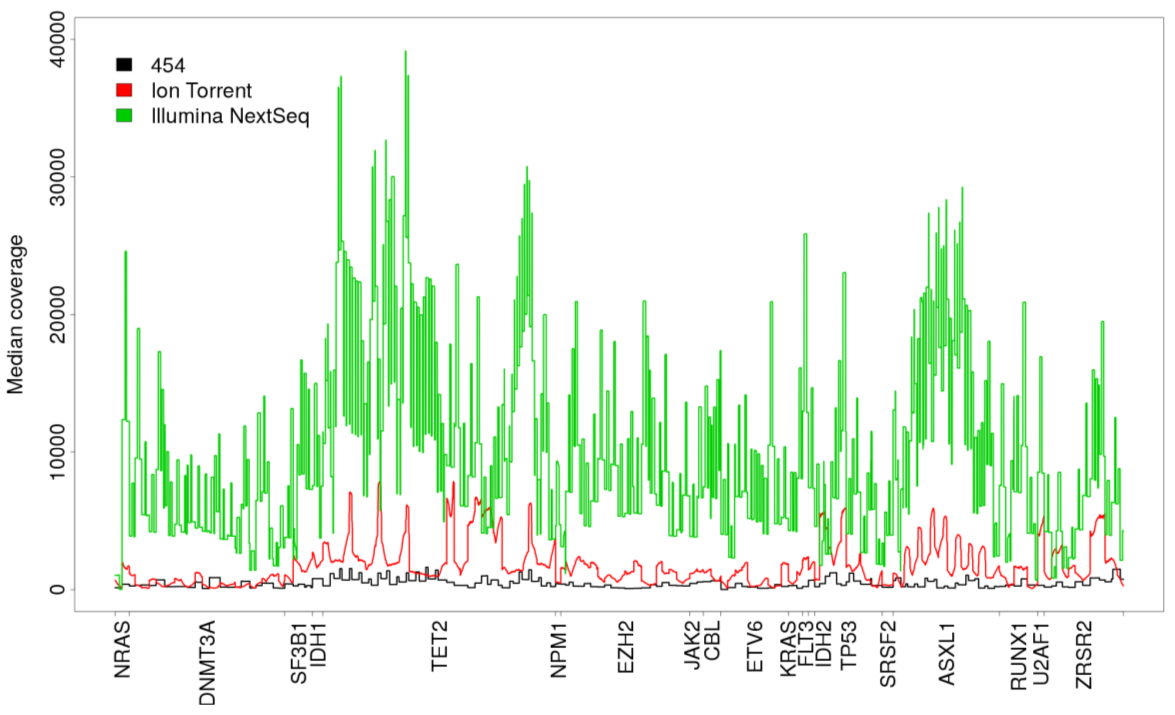

Supplement: S4 Fig — (TIF) [file pone.0171983.s030.tif]

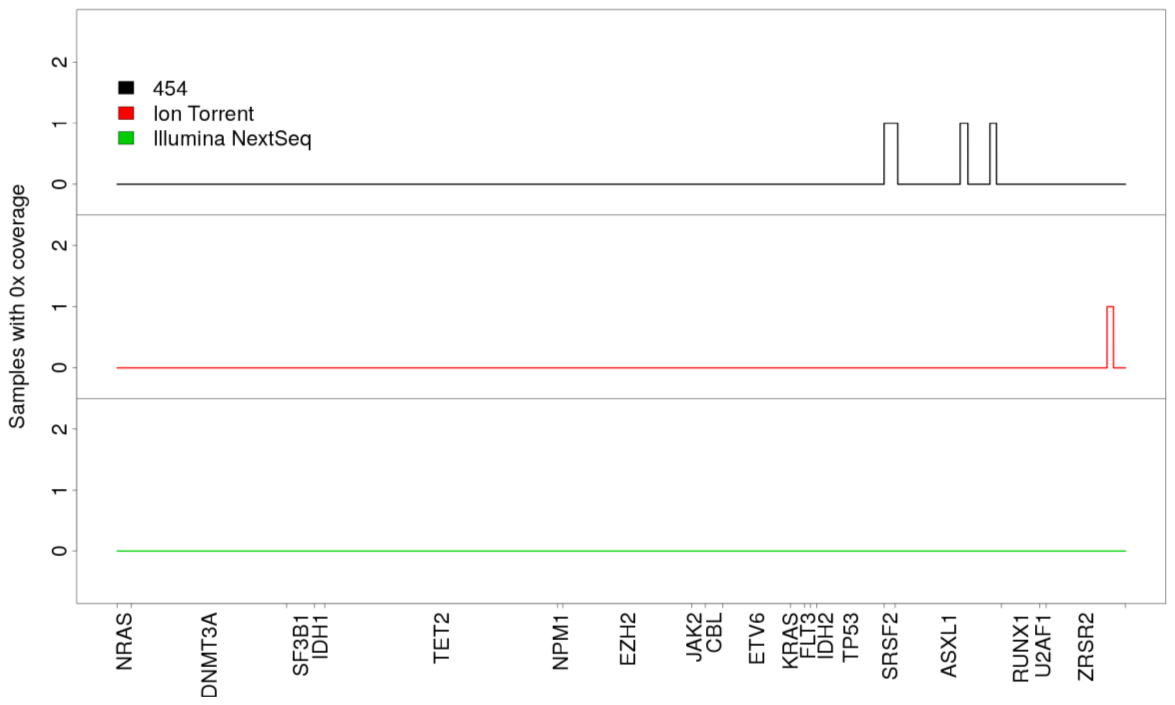

Supplement: S5 Fig — (TIF) [file pone.0171983.s031.tif]

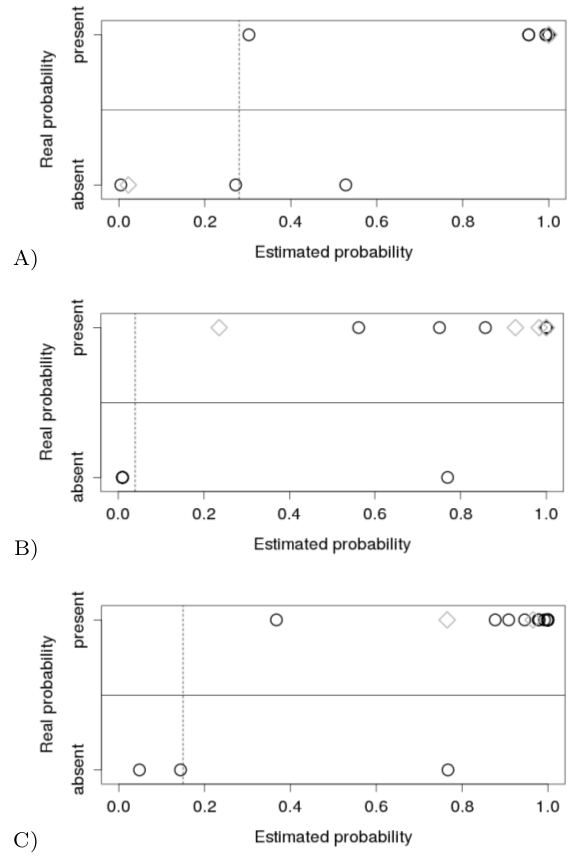

Supplement: S6 Fig — (A) 454 (B) Ion Torrent (C) Illumina; Black circles: training data set; grey diamonds: test data set. Thresholds are displayed as dashed lines (pSNV_454 = 0.28, pSNV_IonT = 0.04 and pSNV_Illumina = 0.07). (TIF) [file pone.0171983.s032.tif]

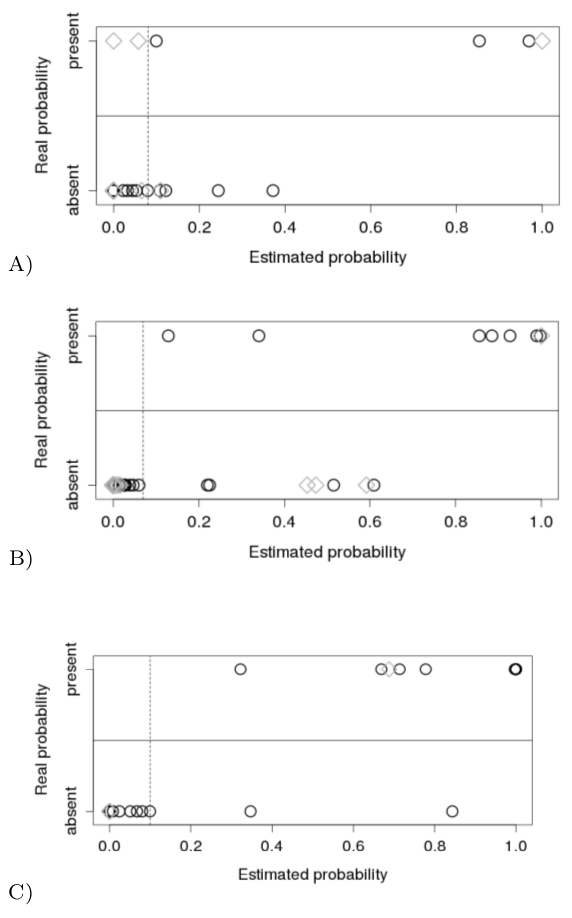

Supplement: S7 Fig — (A) 454 (B) Ion Torrent (C) Illumina; Black circles: training data set; grey diamonds: test data set. Thresholds are displayed as dashed lines (pIndel_454 = 0.08, pIndel_IonT = 0.07 and pIndel_Illumina = 0.01). (TIF) [file pone.0171983.s033.tif]

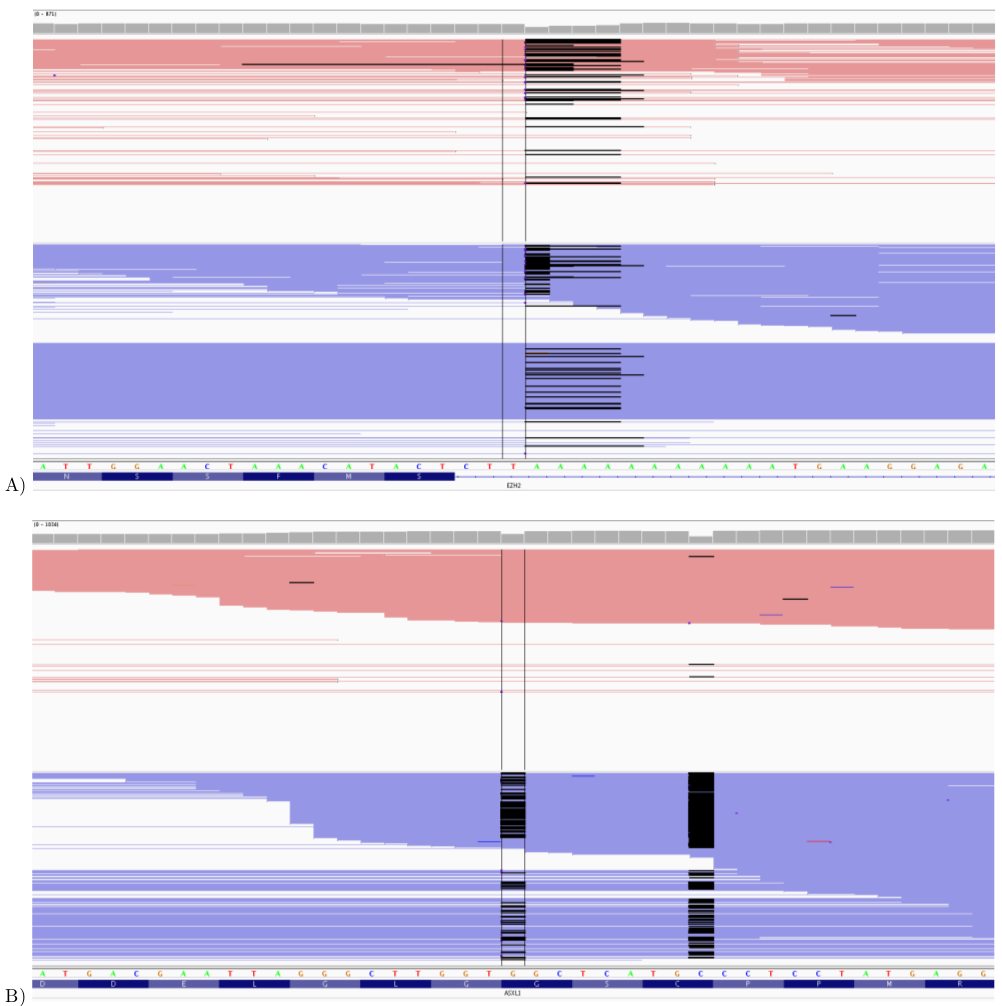

Supplement: S8 Fig — (A) False positive call chr7:148,543,693 TAAAA>T in sample UPN02, Ion Torrent, set2; observed variation in the call (insertion of two A’s up to deletion of five A’s) is strong evidence for a false positive. (B) False positive call chr20:31023122 TG>T in sample UPN007, Ion Torrent, set2; observed strand bias is strong evidence for a false positive. (TIF) [file pone.0171983.s034.tif]
